# Helicobacter pylori upregulates PAD4 expression via stabilising HIF-1α to exacerbate rheumatoid arthritis

**DOI:** 10.1136/ard-2023-225306

**Published:** 2024-08-06

**Authors:** Hui Wu, Hanmei Yuan, Jin Zhang, Taojun He, Yilin Deng, Ying Chen, Yunqi Zhang, Weisan Chen, Chao Wu

**Affiliations:** 1 Department of Laboratory Medicine, The Eighth Affiliated Hospital of Sun Yat-Sen University, Shenzhen, Guangdong, China; 2 Shenzhen Futian Hospital for Rheumatic Diseases, Shenzhen, Guangdong, China; 3 Biochemistry and Genetics, La Trobe University, Melbourne, Victoria, Australia

**Keywords:** Arthritis, Rheumatoid, Anti-Citrullinated Protein Antibodies, Antibodies

## Abstract

**Objective:**

*Helicobacter pylori* infection has been reported to aggravate rheumatoid arthritis (RA), but the relevant mechanism remains unclear. This study aimed to investigate the underlying pathogenic mechanism of *H. pylori* infection in the progression of RA.

**Methods:**

The Disease Activity Score (DAS-28) and serum anticitrullinated protein antibody (ACPA) levels were compared between *H. pylori*-negative and *H. pylori*-positive patients with RA. MH7A cells were stimulated with polyclonal ACPA purified from the peripheral blood of patients with RA. The citrullination levels were assessed by western blot in GES-1 cells and sera. ChIP, luciferase reporter assays, mass spectrometry and ELISA were applied to explore the molecular mechanism of *H. pylori* infection in RA progression.

**Results:**

The DAS-28 and ACPA levels of patients with RA in the *H. pylori*-positive group were significantly higher than those in the *H. pylori*-negative group. Polyclonal ACPA derived from *H. pylori*-positive patients promoted cell proliferation and induced secretion of IL-6 and IL-8. For the first time, we found that *H. pylori* infection induces cellular protein citrullination by upregulating protein arginine deiminase type 4 (PAD4). Furthermore, we confirmed a direct functional binding of hypoxia-inducible factor 1α on the *PADI4* gene promoter. We demonstrated that PAD4 interacts with and citrullinates keratin 1 (K1), and serum and synovial fluid levels of anti-Cit-K1 antibody were markedly increased in *H. pylori*-infected patients with RA.

**Conclusion:**

Our findings reveal a novel mechanism by which *H. pylori* infection contributes to RA progression. Therapeutic interventions targeting *H. pylori* may be a viable strategy for the management of RA.

**WHAT IS ALREADY KNOWN ON THIS TOPIC**

- *Helicobacter pylori* infection has been linked to aggravation of rheumatoid arthritis (RA), but the underlying mechanisms remain understood.

**WHAT THIS STUDY ADDS**

- *H. pylori* infection promotes synovial cell proliferation and inflammation through anticitrullinated protein antibody.
- *H. pylori* infection upregulates the expression of PAD4 through stabilising hypoxia-inducible factor 1α.
- *H. pylori* infection induces PAD4-mediated K1 citrullination; the generated Cit-K1 may induce anti-Cit-K1 antibody production.

**HOW THIS STUDY MIGHT AFFECT RESEARCH, PRACTICE OR POLICY**

- These findings reveal a novel mechanism between *H. pylori* infection and RA pathology.
- Furthermore, incorporating antibiotic treatment of *H. pylori* infection into the standard management protocol for RA warrants consideration in future clinical practice.

## Introduction

 Rheumatoid arthritis (RA) is a chronic autoimmune disease characterised by synovial inflammation that results in permanent joint damage and disability.^1^ The aetiology of RA is multifactorial and usually associated with genotype and environment.^2^ Microbiota is one of the essential environmental factors. Mounting evidence has suggested that microbiota plays a critical role in the development and progression of RA.^3^,^9^

*Helicobacter pylori*is a gram-negative, microaerophilic, spiral-shaped bacterium that infects 50% of the global population and has been classified as a class I human carcinogen.^10^ To date, the link between *H. pylori* infection and RA onset remains controversial.^11 12^ However, it has been well observed that patients with RA with *H. pylori* infection exhibited a propensity for more severe clinical manifestations in comparison to patients with RA without the infection.^13 14^ Additionally, eradicating *H. pylori* effectively improved the clinical outcomes of patients with RA.^14 15^ These findings strongly imply that *H. pylori* infection may be associated with the progression of RA. However, the potential mechanism of such an association is still unclear.

The aberrant production of anticitrullinated protein antibody (ACPA) is a hallmark of RA.^16 17^ ACPA specifically recognise proteins that possess the amino acid citrulline, which is a critical post-translational modification (PTM) catalysed by peptidylarginine deiminases (PADs), thereby triggering complement activation and subsequent release of inflammatory factors.^17 18^ Furthermore, perturbed citrullination can stimulate immune reactions, resulting in the generation of ACPA.^19 20^ Although a direct link between citrullination and microbial infection has emerged,^21 22^ it remains unknown whether *H. pylori* infection could induce citrullination.

In this study, we demonstrate that *H. pylori* infection plays a facilitatory role in the progression of RA through ACPA. Molecular and functional experiments revealed that the PAD4 was upregulated through the reactive oxygen species (ROS)/hypoxia-inducible factor 1α (HIF-1α) signalling pathway, and keratin 1 (K1) was identified as the target protein. Furthermore, we found that patients with RA with *H. pylori* infection exhibited higher levels of anti-citrullinated-K1 (anti-Cit-K1) antibodies in both serum and synovial fluid. Overall, we identify a novel mechanism by which *H. pylori* infection contributes to RA progression, which may provide new insight into therapeutic strategies for RA.

## Methods

Please see online supplemental material.

## Results

### *H. pylori*infection participates in the progression of RA and promotes synovial cell proliferation and inflammation through ACPA

**Figure 1:**
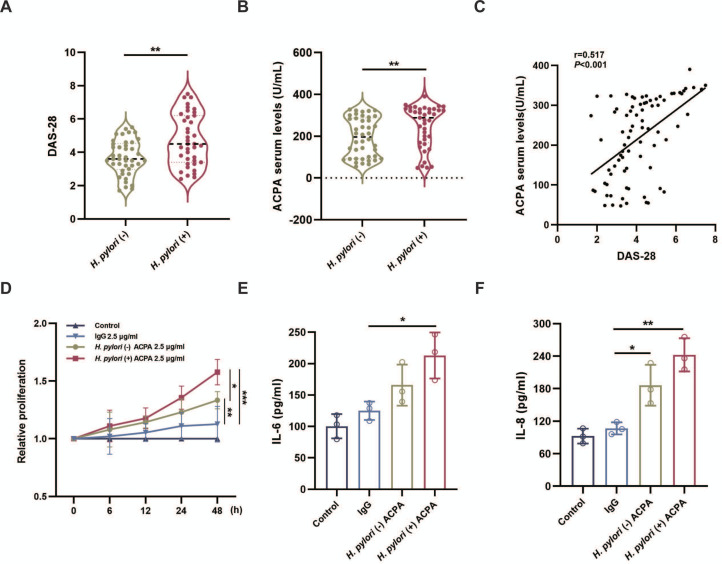
Effect of *Helicobacter pylori* infection on patients with RA and MH7A cells. (**A**) The difference in DAS-28 between *H. pylori*-negative (n=42) and *H. pylori*-positive (n=39) RA groups. (**B**) Comparison of serum ACPA in patients with RA grouped by *H. pylori*. (**C**) Correlation of DAS-28 with ACPA. (**D**) MH7A cells were treated with 2.5 µg/mL ACPA IgG (ACPA) or non-ACPA control IgG (IgG) or without any antibody (Control) for indicated times (6 hour, 12 hours, 24 hours, 48 hours), followed by cell proliferation analyses using CCK8 assay. (**E, F**) IL-6 and IL-8 levels in supernatants were measured after stimulating with 2.5 µg/mL ACPA or IgG for 48 hours. Statistical analyses were performed using the Student’s t-test (**A**), Mann-Whitney U test (**B**), two-way ANOVA (**D**) and one-way ANOVA (**E, F**). (**A, B**) Data are presented as violin plots with the median (black dotted line) and quartiles (green and red dotted lines) indicated. (**D–F**) Data are presented as mean±SD. *p<0.05, **p<0.01, ***p<0.001. ACPA, anticitrullinated protein antibody; ANOVA, analysis of variance; DAS-28, Disease Activity Score 28; RA, rheumatoid arthritis.

We first analysed the Disease Activity Score 28 (DAS-28) in 39 *H. pylori*-infected and 42 uninfected patients with RA and found that DAS-28 values were significantly higher in *H. pylori*-positive patients with RA than in *H. pylori*-negative patients with RA (figure 1A), suggesting that *H. pylori* infection might be associated with RA exacerbation.

Given the high specificity of ACPA for RA, we next assessed the serum levels of ACPA.

Compared with *H. pylori*-negative patients with RA, *H. pylori*-positive patients with RA exhibited significantly elevated serum ACPA levels (figure 1B). Furthermore, there was a significant positive correlation between serum ACPA levels and DAS-28 (figure 1C).

Fibroblast-like synoviocytes (FLSs) are essential effector cells in RA that contribute to the pathological process of synovial membrane hyperplasia and inflammation.^23^ It has been reported that FLSs are sensitive to ACPA.^24^ Therefore, we explored whether *H. pylori* infection affects FLSs through ACPA. Two cohorts of affinity-purified ACPA IgGs were obtained from patients with RA with or without *H. pylori* infection (n1=39, n2=42). We found that ACPA IgG, as opposed to control IgGs (non-binding CCP IgGs), exhibited notable efficacy in inducing MH7A cell proliferation (figure 1D). ELISA experiments revealed a significant increase in IL-6 and IL-8 levels in the culture supernatants of MH7A cells treated with ACPA from *H. pylori*-positive patients (figure 1E,F). These findings indicate that *H. pylori* infection may affect synovial pathology through ACPA.

### *H. pylori* infection promotes protein citrullination

**Figure 2:**
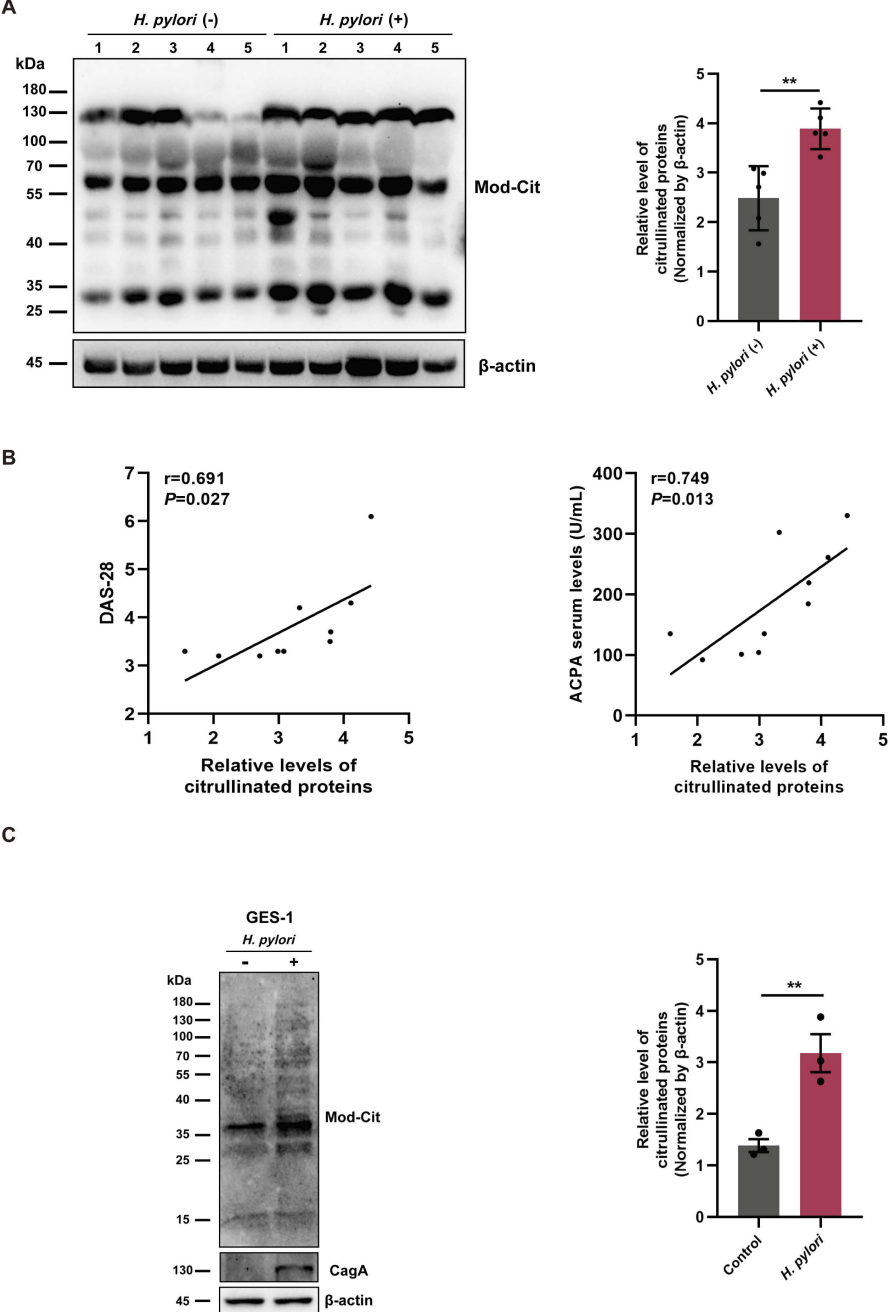
Detection of citrullinated proteins. (**A**) Western blot analysis of citrullinated proteins in the serum of patients with RA infected with or without *Helicobacter pylori* (n=5 each). (**B**) Correlation of serum levels of citrullinated proteins with DAS-28 and ACPA in patients with RA. (**C**) Western blot analysis of citrullinated proteins in GES-1 cells infected with the *H. pylori* NCTC 11637 strain (MOI=100) for 24 hours. The cell experiment was independently repeated three times. Data are presented as mean±SD. Statistical analyses were performed with the Student’s t-test. **p<0.01. ACPA, anticitrullinated protein antibody; DAS-28, Disease Activity Score 28; MOI, multiplicities of infection; RA, rheumatoid arthritis.

Citrullinated proteins are a prerequisite for generating ACPA.^25^ Thus, we hypothesised that citrullination levels would be increased during *H. pylori* infection. Using an antimodified citrulline antibody, immunoblot analysis showed that serum citrullinated proteins were markedly increased in the *H. pylori*-infected group (figure 2A). Importantly, we found a strong positive correlation between citrullinated protein levels and DAS-28 or ACPA (figure 2B), indicating that elevated citrullination corresponds with increased disease activity and ACPA levels. To further determine the effect of *H. pylori* infection on protein citrullination, we analysed citrullinated protein levels in GES-1 cells infected with standard *H. pylori* strain NCTC 11637. Consistent with the above serological data, citrullinated proteins were significantly elevated in *H. pylori*-infected GES-1 cells compared with uninfected control cells (figure 2C). Together, these data suggest that *H. pylori* infection promotes protein citrullination.

### PAD4 is crucial for *H. pylori*-induced citrullination

**Figure 3:**
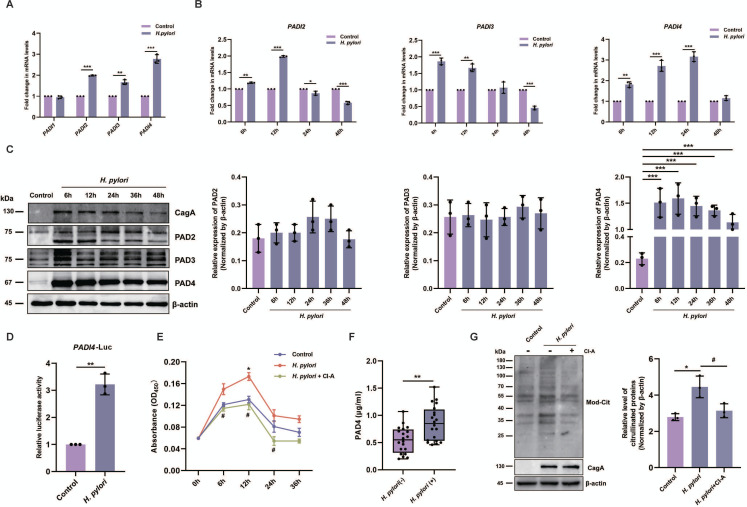
*Helicobacter pylori* infection induces PAD4 expression and enhances enzyme activity. (**A**) RT-qPCR analysis of *PADI* isoform mRNA expression levels after *H. pylori* infection (MOI=100) in GES-1 cells for 12 hours. (**B**) *PADI2*, *PADI3* and *PADI4* mRNA levels in *H. pylori*-infected GES-1 cells (MOI=100) at different time points. (**C**) Western blot analysis of PAD2, PAD3 and PAD4 at 6, 12, 24, 36 and 48 hours after *H. pylori* infection (MOI=100) in GES-1 cells. (**D**) Dual-luciferase reporter assay of *PADI4* promoter activities in GES-1 cells infected with *H. pylori* for 12 hours. (**E**) Detection of PAD4 enzymatic activity in *H. pylori-*infected (MOI=100) or uninfected (control) GES-1 cells in the presence (green line) or absence (untreated or *H. pylori* alone, blue and red lines, respectively) of CI-A (200 µM). (**F**) ELISA assay of PAD4 protein levels in the serum of patients with RA (n=20 each). (**G**) Western blot analysis of citrullinated proteins in GES-1 cells after infection with or without *H. pylori* (MOI=100) for 24 hours in the presence or absence of CI-A (200 µM). Data are presented as mean±SD. Statistical analyses were performed with the Student’s t-test and one-way ANOVA. *p<0.05, **p<0.01, ***p<0.001 vs control group or *H. pylori* (−) group; #p<0.05 vs *H. pylori* group. ANOVA, analysis of variance; MOI, multiplicities of infection.

Citrullination is catalysed by the protein arginine deiminase (PAD) family, containing five isoforms (PAD1-4 and PAD6). To investigate whether PADs play a critical role in enhanced citrullination during *H. pylori* infection, we first assessed the transcriptional level of each PAD gene by RT-qPCR. In GES-1 cells infected with *H. pylori* for 12 hours compared with untreated control cells, *PADI2*, *PADI3* and *PADI4* genes were significantly induced (figure 3A). Moreover, these three genes were notably upregulated after stimulation, reaching peak expression at 6 hours (*PADI3*), 12 hours (*PADI2*) and 24 hours (*PADI4*), respectively (figure 3B). However, the *PADI1* gene expression was not altered following *H. pylori* infection, and *PADI6* remained undetectable (figure 3A). Western blot analysis demonstrated a dramatic increase in PAD4 expression after *H. pylori* infection in GES-1 cells (figure 3C). By contrast, the expression levels of other PAD isoforms were either not altered (PAD2 and PAD3, figure 3C) or not detected (PAD1, online supplemental figure 2B). To validate the regulatory mechanism of *H. pylori* on PAD4, a luciferase reporter plasmid containing the wild-type promoter of *PADI4* was constructed and transfected into GES-1 cells. As shown in figure 3D, *H. pylori* infection caused a robust induction (around 3.5-fold) of the luciferase activity. Next, PAD4 enzymatic activity was measured by an ABAP assay.^26^ Compared with the control, PAD4 catalytic activity was enhanced at 6 hours, peaked at 12 hours, and then sharply decreased (figure 3E). Notably, *H. pylori*-induced PAD4 activity was significantly suppressed by CI-amidine (CI-A, 200 µM, a PAD inhibitor) at 6 hours, 12 hours and 24 hours after *H. pylori* infection (figure 3E). In line with the western blot results (figure 3C), a significant increase in the level of PAD4 secretion was observed in sera from *H. pylori*-infected patients compared with those from their counterparts (figure 3F). Additionally, protein citrullination was considerably inhibited in the presence of CI-A (figure 3G). These results indicate that *H. pylori* infection facilitates citrullination by upregulating PAD4 expression.

### *H. pylori* infection upregulates PAD4 through ROS/HIF-1α axis

**Figure 4:**
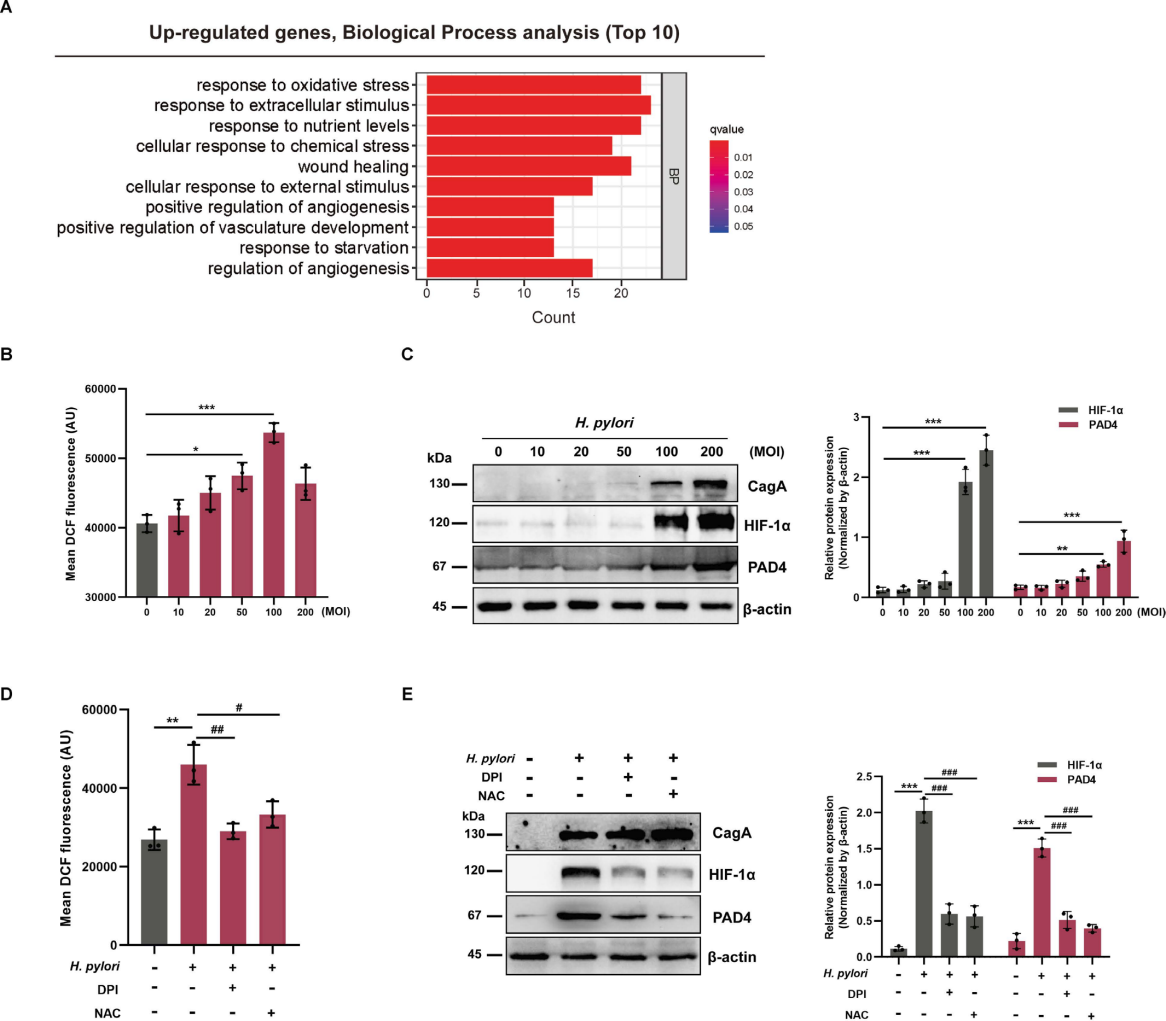
*Helicobacter pylori* infection regulates PAD4 expression in an ROS-dependent manner. (**A**) A bar chart showing the top 10 enriched pathways in upregulated genes of *H. pylori*-infected GES-1 cells compared with uninfected cells. (**B**) Detection of ROS levels in GES-1 cells with or without *H. pylori* infection (MOI=0, 10, 20, 50, 100 and 200) for 6 hours. (**C**) Western blot analysis of HIF-1α and PAD4 in GES-1 cells infected with different MOIs of *H. pylori* for 6 hours. (**D**) Detection of ROS levels in GES-1 cells treated with ROS inhibitors DPI (100 µM) or NAC (20 mM) for 1 hour prior to *H. pylori* infection (MOI=100, 6 hours). (**E**) Western blot analysis of HIF-1α and PAD4 in GES-1 cells pretreated with DPI (100 µM) or NAC (20 mM) for 1 hour followed by *H. pylori* (MOI=100) treatment for 6 hours. Data are presented as mean±SD. Statistical analyses were performed with a one-way ANOVA. *p<0.05, **p<0.01, ***p<0.001 vs MOI=0 group; #p<0.05, ##p<0.01, ###p<0.001 vs *H. pylori* group. ANOVA, analysis of variance; DPI, diphenyleneiodonium chloride; MOI, multiplicities of infection; NAC, N-Acetylcysteine; ROS, reactive oxygen species.

To explore the signalling pathway that regulates PAD4 expression by *H. pylori*, we performed a Gene Ontology (GO) enrichment analysis using GES-1 cells with or without *H. pylori* infection from the GEO public database (GSE74577). Our analysis demonstrated that upregulated genes are mainly involved in stress responses, particularly oxidative stress (OS) (figure 4A). Excessive ROS is a prominent feature of OS, and it plays an essential role in stabilising HIF-1.^27 28^ Earlier reports showed that *H. pylori* increased HIF-1α levels via ROS-dependent mechanisms.^29 30^ Similar to the previously reported results, we found dramatically higher ROS levels in GES-1 cells at 6 hours postinfection with *H. pylori* (MOI=50, 100; figure 4B). In addition, the expression of HIF-1α and PAD4 was upregulated in GES-1 cells following infection with *H. pylori* for 6 hours in an MOI-dependent manner (figure 4C). To investigate whether infection with other bacteria could have a similar effect, we infected GES-1 cells with two intestinal bacteria, *Escherichia coli* and *Prevotella copri*. Neither *E. coli* nor *P. copri* infection had any effect on ROS levels or HIF-1α expression (online supplemental file 3A–D). Interestingly, PAD4 was significantly increased in an MOI-dependent manner following *E. coli* infection (online supplemental figure 3C). These results indicate that the ROS-mediated regulation of PAD4 is specific for *H. pylori* infection.

Furthermore, we defined whether the ROS/HIF-1α axis participated in regulating PAD4. GES-1 cells were pretreated with ROS inhibitors, diphenyleneiodonium chloride (DPI, 100 µM) or N-acetylcysteine (NAC, 20 mM) for 1 hour and then infected with *H. pylori* for 6 hours. The intracellular ROS levels induced by *H. pylori* were significantly decreased in the presence of DPI or NAC (figure 4D). Moreover, we found that using DPI or NAC strongly reduced the *H. pylori-*induced increases of HIF-1α and PAD4 (figure 4E), suggesting that *H. pylori-*induced PAD4 expression depended on ROS/HIF-1α signalling pathway.

### HIF-1α binds to *PADI4* promoter and modulates *PADI4* expression

**Figure 5:**
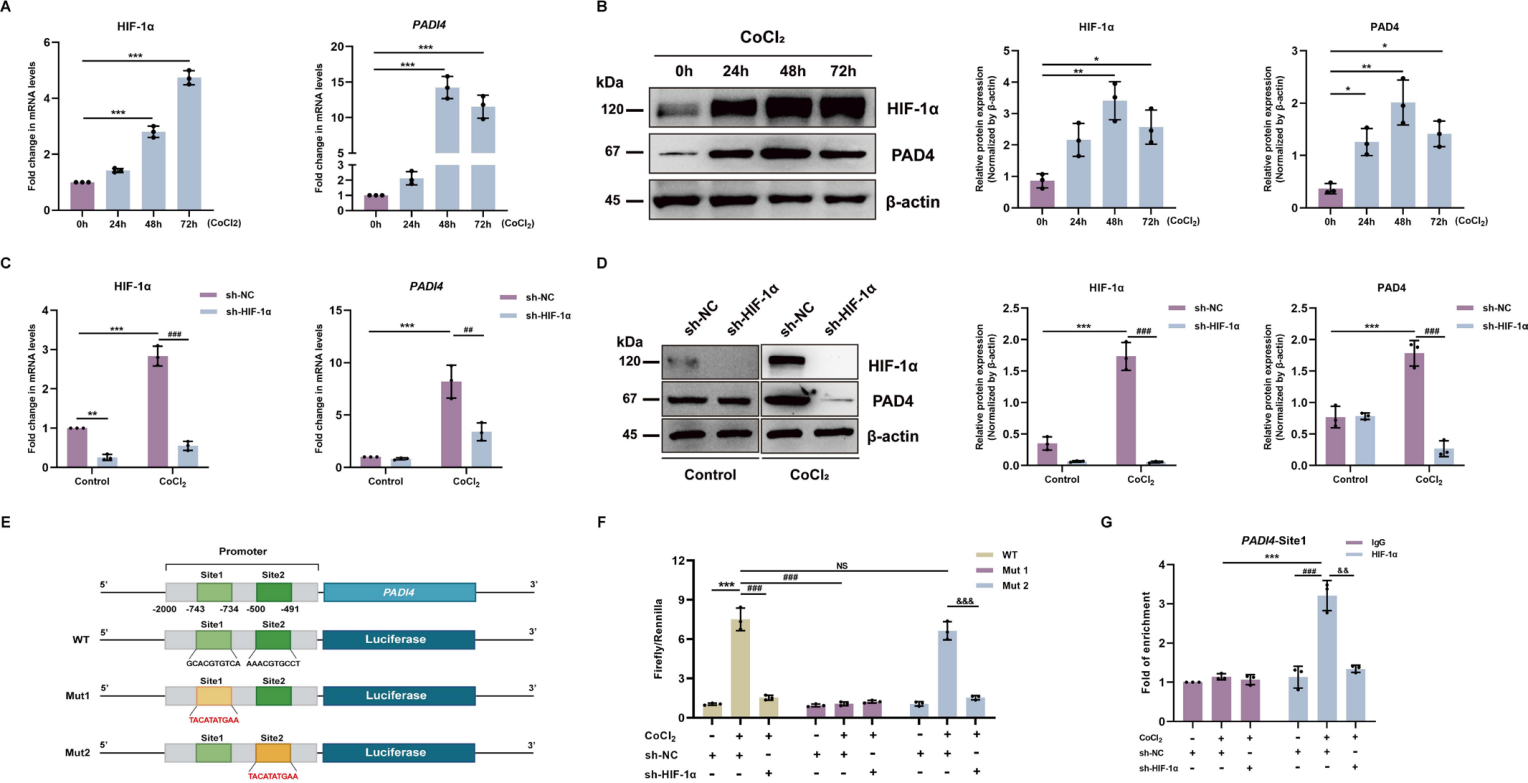
HIF-1α directly binds to the *PADI4* promoter and initiates gene transcription. (**A**) RT-qPCR analysis of HIF-1α and *PADI4* mRNA expression levels after CoCI_2_ treatment (100 µM) at 0 hour, 24 hours, 48 hours and 72 hours in GES-1 cells. (**B**) Western blot analysis of HIF-1α and PAD4 in GES-1 cells treated with CoCI_2_ (100 µM) for different time points. (**A, B**) *p<0.05, **p<0.01, ***p<0.001 vs control group (time point=0 hour). (**C**) RT-qPCR analysis of HIF-1α and *PADI4* mRNA expression levels in lentiviral shRNA HIF-1α transfected GES-1 cells following CoCI_2_ treatment (100 µM, 48 hours). (**D**) Western blot analysis of HIF-1α and PAD4 in HIF-1α knockdown GES-1 cells treated with CoCI_2_ (100 µM, 48 hours). (**C, D**) **p<0.01, ***p<0.001 vs control group transfected with sh-NC; ##p<0.01, ###p<0.001 vs CoCI_2_ treatment group transfected with sh-NC. (**E**) A schematic diagram of firefly luciferase constructs containing the *PADI4* promoter, showing the wild-type sequence (WT) and the mutation of the HIF-1α binding sites (Mut). Site 1: −743 to −734 bp; site 2: −500 to −491 bp. (**F**) *PADI4* promoter luciferase reporter assay was performed in GES-1 cells with HIF-1α depletion followed by CoCI_2_ treatment (100 µM, 48 hours). ***p<0.001 vs WT+sh NC group; NS, ###p<0.001 vs WT+CoCI_2_+sh NC group; &&&p<0.001 vs Mut2+CoCI_2_+sh NC group. (**G**) ChIP assay using HIF-1α antibody was performed, followed by RT-qPCR using the primer covering the site1 region. ***p<0.001 vs IgG antibody+CoCI_2_+sh NC group; ###p<0.001 vs HIF-1α antibody+sh NC group; &&p<0.01 vs HIF-1α antibody+CoCl_2_+sh NC group. Data are presented as mean±SD. Statistical analyses were performed with a one-way ANOVA. ANOVA, analysis of variance; HIF-1α, hypoxia-inducible factor 1-alpha; NS, no significant difference.

Based on the above results, we hypothesised that HIF-1α might regulate PAD4. To verify this, we used cobalt chloride (CoCl_2_, 100 µM), a hypoxia-mimetic agent, to activate HIF-1α in GES-1 cells, which led to more than 10-fold induction of *PADI4* mRNA expression at 48 hours and 72 hours (figure 5A), as well as 3–4 fold increase in PAD4 protein levels at 24 hours, 48 hours and 72 hours (figure 5B). To further prove that HIF-1α is the upstream regulator of PAD4, we knocked down HIF-1α expression by transfecting HIF-1α sh-RNA lentivirus in GES-1 cells before treating them with CoCl_2_ for 48 hours. The HIF-1α knockdown markedly suppressed PAD4 mRNA and protein expression induced by CoCI_2_ (figure 5C,D).

Given that the HIF-1α protein can activate the transcription of target genes via direct binding to their promoter,^31^ we wondered whether PAD4 is a direct target of HIF-1α. We then analysed the *PADI4* promoter using the JASPAR database (http://jaspar.genereg.net) and identified two highly possible HIF-1α binding sites in the *PADI4* promoter region (figure 5E, green bars). To investigate whether these two sites could function as hypoxia response elements (HREs), we designed a mutation in either site of the *PADI4* promoter sequence (figure 5E, yellow bars). Overexpression of HIF-1α by CoCI_2_ treatment increased *PADI4* promoter luciferase activity (figure 5F). Knocking down HIF-1α or mutating the binding site 1 inhibited the luciferase induction by CoCI_2_ treatment (figure 5F). However, mutating binding site 2 failed to alter the luciferase activity induced by HIF-1α (figure 5F). To further confirm that site 1 was the HIF-1α binding sequence, we designed a primer covering binding site 1 for a chromatin immunoprecipitation (ChIP) assay. After treatment with CoCI_2_, the HIF-1α antibody precipitated DNA fragments containing site 1 compared with the IgG control (figure 5G). Moreover, silencing HIF-1α led to a significant decrease in the occupancy of the *PADI4* HRE (figure 5G). These results indicate that HIF-1α binds directly to the *PADI4* promoter and activates *PADI4* gene transcription.

### 6. PAD4 citrullinates K1 protein during *H. pylori* infection

**Figure 6:**
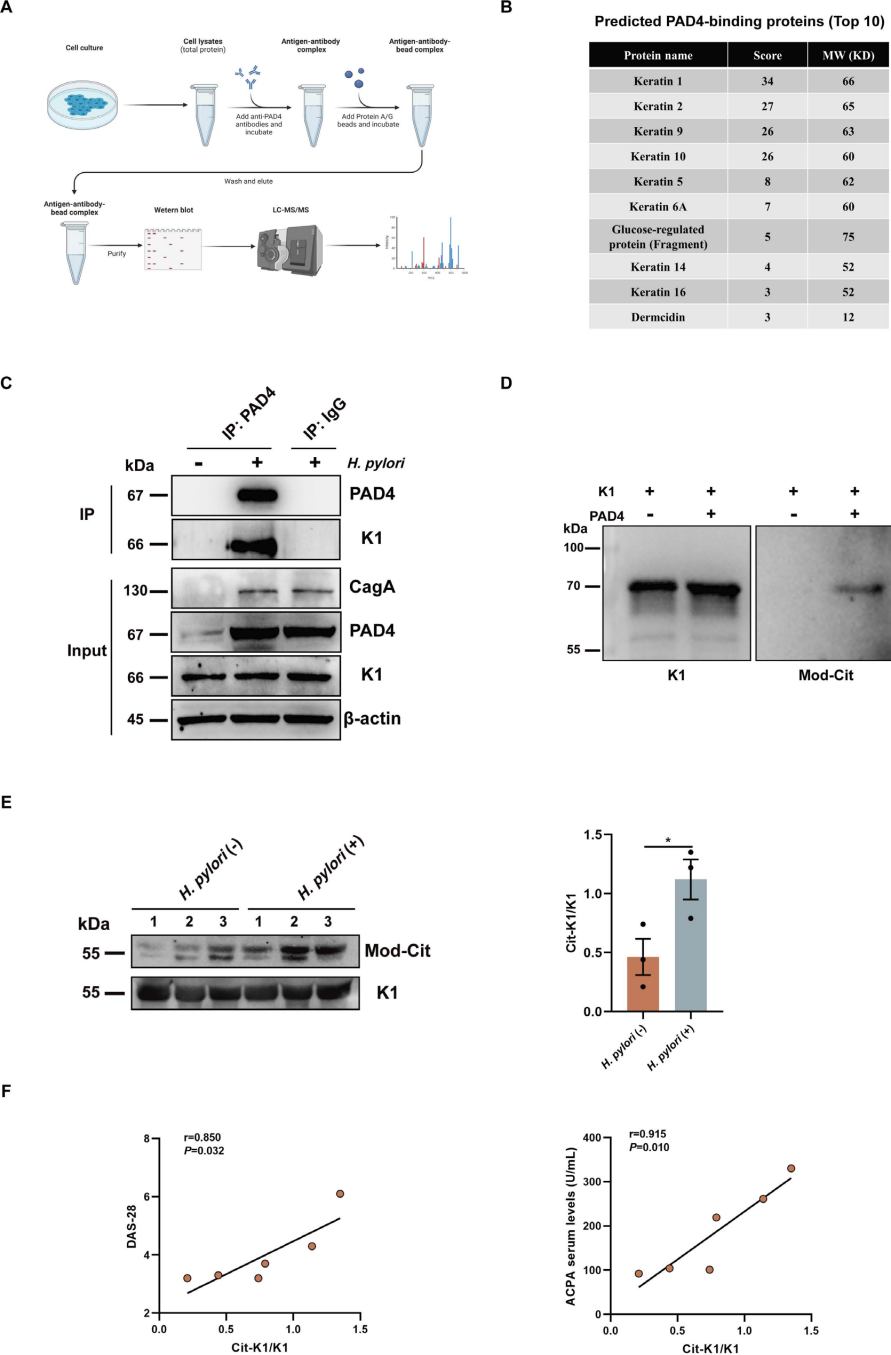
Identification of K1 as a downstream target protein of PAD4. (**A**) Schematic diagram of the pull-down experiment. (**B**) MS identification of PAD4-interaction proteins in GES-1 cells infected with *H. pylori*. The value of unique peptides represents the score. (**C**) Immunoprecipitation analysis of the interaction between PAD4 and K1. (**D**) Citrullination of K1 by PAD4 in vitro. The reactions were assessed by western blot using anti-K1 and anti-modified citrulline antibodies. (**E**) Expression of citrullinated K1 (Cit-K1) in serum samples from patients with RA infected with or without *H. pylori* (n=3 each). Cit-K1 was analysed by western blot using anti-K1 and anti-modified citrulline antibodies. (**F**) Correlation of serum levels of Cit-K1 with DAS-28 and ACPA in patients with RA. All cell experiments were independently repeated three times. Data are presented as mean±SD. Statistical analyses were performed with the Student’s t-test. *p<0.05. ACPA, anticitrullinated protein antibody; DAS28, Disease Activity Score 28; RA, rheumatoid arthritis.

To search for PAD4 binding proteins, we performed a pull-down experiment using a PAD4-specific antibody. PAD4-binding protein in GES-1 cells with or without *H. pylori* infection for 6 hours was analysed by LC-MS/MS analysis (figure 6A, online supplemental file 5). Keratin 1 (K1) was identified as the most prominent PAD4-interacting protein (figure 6B). Immunoprecipitation assays confirmed that K1, but not Keratin 2 (K2), Keratin 9 (K9) or Keratin 10 (K10), was bound to PAD4 (figure 6C, online supplemental figure 6). To further define K1 as a PAD4 substrate, His-tagged K1 was puriﬁed and incubated with recombinant PAD4. Then, the dissolved proteins were analysed with an anti-modified citrulline monoclonal antibody (anti-MC mAb). Results demonstrated that the antimodified citrulline antibody specifically recognised only K1 treated with PAD4 (figure 6D), suggesting that K1 is a target for PAD4-mediated citrullination.

To evaluate the citrullinated K1 (Cit-K1) proteins in sera from patients with RA, we performed immunoprecipitation with an anti-K1 antibody and western blot with anti-K1 and anti-modified citrulline antibodies. Proteins of approximately 55 kDa from these sera reacted with the anti-K1 and anti-modified citrulline antibodies, confirming that the citrullinated protein was Cit-K1. Cit-K1 levels in the *H. pylori*-positive serum samples were dramatically higher than those in the *H. pylori*-negative serum samples (figure 6E). Furthermore, we observed an association between Cit-K1 levels and DAS-28 and ACPA (figure 6F). These findings demonstrate that PAD4 catalyses the citrullination of K1, and Cit-K1 may facilitate RA pathogenesis.

### Detection of anti-CIT-K1 antibody in patient with RA

**Figure 7:**
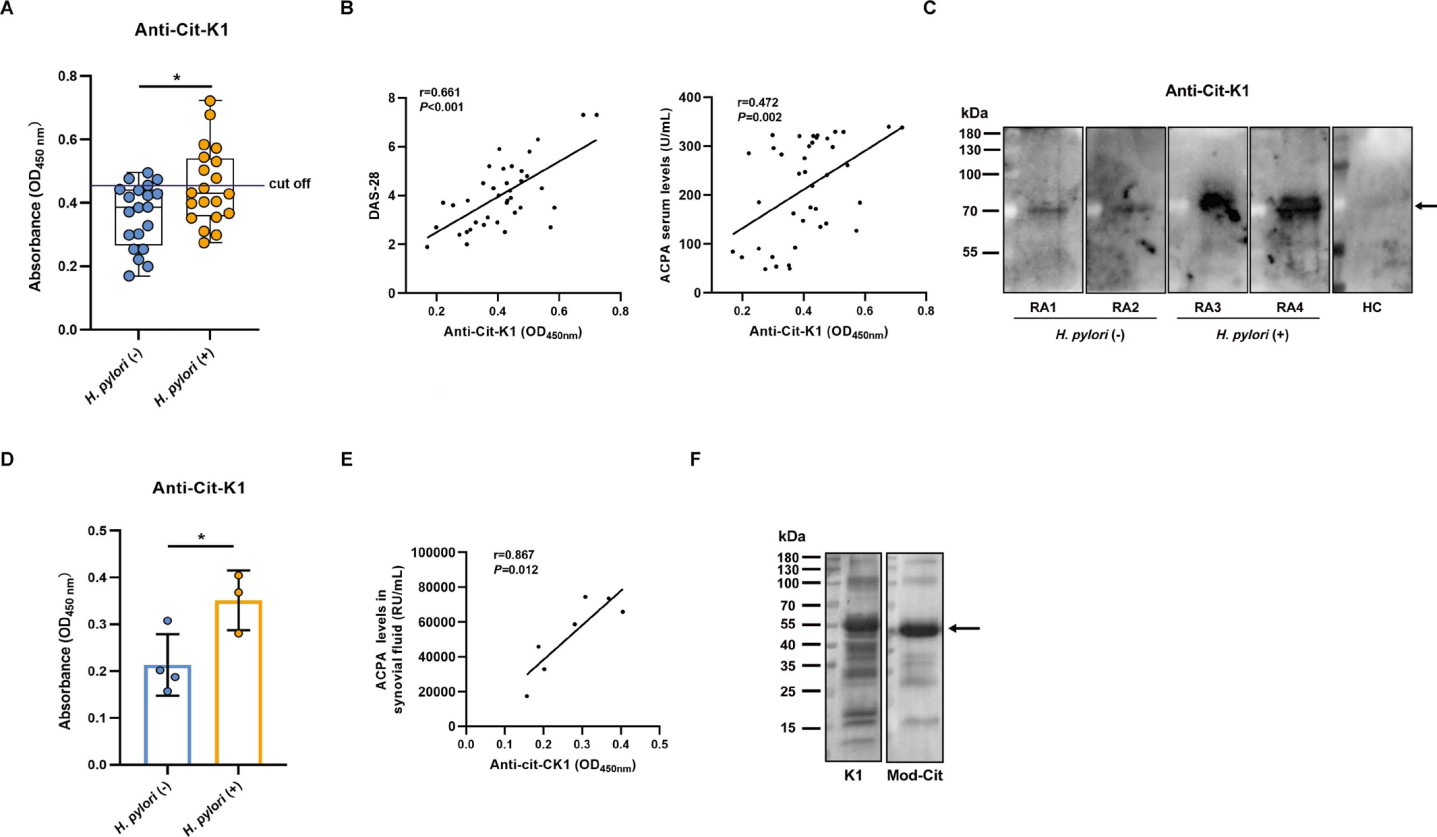
Detection of anti-Cit-K1 antibody. (**A**) Measurement of anti-Cit-K1 antibody by ELISA in sera of patients with RA with or without *Helicobacter pylori* infection (n=20, each group). The black line indicates the assay cut-off value (OD_450nm_=0.45). Data are shown as box plots, where the box line represents the median, and the error bars represent the minimum and maximum values. (**B**) Correlation of anti-Cit-K1 antibody levels with DAS-28 and ACPA. (**C**) Western blot for detecting anti-Cit-K1 antibody in serum samples from *H. pylori* (−) RA, *H. pylori* (+) RA and healthy control (HC). (**D**) Anti-Cit-K1 antibody levels in the synovial fluid of *H. pylori*-negative and *H. pylori*-positive patients with RA (n1=4, n2=3) were assessed by ELISA. Data are presented as mean±SD. (**E**) Correlation between anti-Cit-K1 antibody levels and ACPA in synovial fluid. (**F**) Expression of Cit-K1 in synovial tissue of patient with RA (n=1). Cit-K1 was analysed by western blot using anti-K1 and anti-modified citrulline antibodies. Statistical analyses were performed using the Student’s t-test. *p<0.05. ACPA, anticitrullinated protein antibody; DAS-28, Disease Activity Score 28; RA, rheumatoid arthritis.

Given that citrullinated proteins induce ACPA production, we speculate that Cit-K1 would elicit its specific antibodies. Therefore, serum samples from 40 patients with RA (20 *H*. *pylori*-negative, 20 *H*. *pylori*-positive) were analysed for the anti-Cit-K1 antibody by ELISA. *H. pylori*-positive patients with RA exhibited significantly higher anti-Cit-K1 antibody levels than those from *H. pylori*-negative patients (figure 7A). In addition, anti-Cit-K1 antibody levels were positively associated with Cit-K1 (online supplemental figure 7), DAS-28 and ACPA (figure 7B). Moreover, the anti-Cit-K1 antibody-positive sera demonstrated in ELISA were confirmed by western blots. Consistent with the ELISA findings, a distinct positive band was detected in these sera, but not in those from healthy controls (figure 7C). We further measured the levels of anti-Cit-K1 antibody in the synovial fluid of patients with RA (four *H*. *pylori*-negative and three *H*. *pylori*-positive). ELISA analysis demonstrated that the anti-Cit-K1 antibody in the *H. pylori*-positive patients was significantly increased, compared with that in the *H. pylori*-negative patients (figure 7D). There was also a positive correlation between synovial fluid levels of anti-Cit-K1 antibody and ACPA (figure 7E). To confirm the Cit-K1 expression in synovial membrane, we collected the synovial lining tissue from the sole patient with RA and performed western blots using antibodies against K1 and modified citrulline. The expression of Cit-K1 was observed as a 55 kDa band in synovial membrane (figure 7F). In conclusion, these results suggest that *H. pylori* infection contributes to RA progression by citrullinating K1, which may lead to Cit-K1 autoantibody production.

## Discussion

RA is among the most common chronic inflammatory diseases, affecting around 1% of the global population.^1^ *H. pylori*, a prevalent bacteria in humans, has been intensively studied over the past three decades to identify the characteristics responsible for instigating host immunity that results in some autoimmune diseases.^32^,^34^ In this study, we demonstrated that disease activity and ACPA levels were significantly higher in *H. pylori*-infected patients with RA than in *H. pylori*-uninfected patients with RA, which is consistent with a recent study.^13^ In addition, ACPA levels were correlated positively with disease activity. The abnormal proliferation of FLSs is regarded as a pathological characteristic of RA. Our study revealed that ACPA IgGs from *H. pylori*-positive patients with RA have a significant effect on facilitating the proliferation of MH7A cells and the secretion of inflammatory factors (IL-6 and IL-8). These results indicate that *H. pylori* infection participates in RA progression in an ACPA-mediated manner.

It is well known that ACPA generation is induced by citrullinated antigens.^35^ The current study showed that citrullinated proteins were remarkably increased in sera and cells from the *H. pylori*-infected group compared with the uninfected group, and expression levels positively correlated with ACPA levels, which supports the hypothesis that *H. pylori* infection may increase ACPA production by promoting citrullination. Citrullination is an irreversible PTM catalysed by PAD enzymes (PAD1-PAD4 and PAD6). Human cytomegalovirus has been shown to induce host protein citrullination by upregulating PAD2 and PAD4.^22^ In the present study, we found that *H. pylori* infection significantly promoted PAD4 expression in GES-1 cells. Intriguingly, the mRNA level of *PADI4* was inconsistent with the protein levels, potentially caused by *H. pylori-*induced DNA damage that could influence gene transcription and pre-mRNA maturation.^36^ In addition, citrullination levels were effectively suppressed in the presence of the PAD inhibitor CI-A. However, the molecular mechanism responsible for regulating PAD4 is still not fully understood.

GO enrichment analysis demonstrated that *H. pylori* infection activates the OS pathway. In line with previously reported results,^29 30^ we observed a remarkable increase in ROS and HIF-1α levels in *H. pylori*-infected GES-1 cells. Inhibiting ROS decreased the *H. pylori*-mediated expression of HIF-1α and PAD4. Similar to *H. pylori* infection, CoCI_2_ treatment induced the expression of PAD4. Knockdown of HIF-1α dramatically reduced the expression of PAD4 both at the transcriptional and protein levels. As a transcriptional regulator, HIF-1α can translocate to the nucleus, form a heterodimer with HIF-1β, and bind to specific DNA sequences in their promoter regions to activate downstream target genes.^37^ The dual-luciferase assay and ChIP assay confirmed HIF-1α binding sites in the PAD4 promoter, supporting the role of HIF-1α in the transcription regulation of PAD4.^38^ Although we unveil a novel mechanism by which *H. pylori* infection upregulates PAD4 by stabilising HIF-1α, we cannot exclude the possibility that other mechanism(s) may be involved in the PAD4 regulation.

PAD enzymes can citrullinate various proteins, such as vimentin, fibrinogen and histones.^39 40^ By employing protein pull-down assay, mass spectrometry analysis and immunoprecipitation, we have identified the ability of *H. pylori* infection to induce citrullination of K1. K1 is a type II keratin family member and serves as a crucial constituent of intermediate filaments within epithelial cells. It is also expressed on the cellular membrane of diverse cancer types.^41^ Moreover, we found significantly higher levels of Cit-K1 in patients with RA with *H. pylori* infection than those without the infection. The molecular weight of Cit-K1 was detected to be approximately 55 kDa, which aligns with the discovery made by Sakaguchi *et al*, who observed a 55 kDa citrullinated protein in the sera of mice with collagen-induced arthritis (CIA).^42^ Although the authors did not confirm the protein further, the molecular weight similarity between Cit-K1 and the protein identified by Sakaguchi *et al* suggests that they may be the same protein.

*H. pylori*-infected patients with RA exhibited notably higher levels of the anti-Cit-K1 antibody both in serum and synovial fluid than uninfected patients with RA. Additionally, the serum anti-Cit-K1 antibody was positively correlated with Cit-K1 expression. These findings indicate that *H. pylori* infection may promote anti-Cit-K1 production through Cit-K1. Anti-keratin antibody (AKA), which recognises the protein filaggrin in rat oesophageal mucosal cells, has also been reported in RA.^43^ However, AKA has lower sensitivity than ACPA, which limits its diagnostic efficacy in RA.^44^ Since K1 is an epithelial cell surface protein, our study further demonstrates the expression of Cit-K1 in synovial membrane from a patient with RA, which is consistent with previous studies.^45 46^ These data, plus the detection of Cit-K1 specific antibody from synovial fluid, suggest the possibility that the anti-Cit-K1 antibody may trigger a local inflammatory response by recognising and binding to its antigen.

There are several limitations in our study. The specific locations of PAD4-dependent citrullination on K1 remain unidentified. Our experiment showed that Cit-K1 expression in synovial membrane was only performed on a single sample due to unforeseen challenge of acquiring such specimens. Further studies should focus on the potential function of Cit-K1 and anti-Cit-K1 antigen-antibody complexes, which could shed new light on the mechanisms of relevant pathologies.

**Figure 8:**
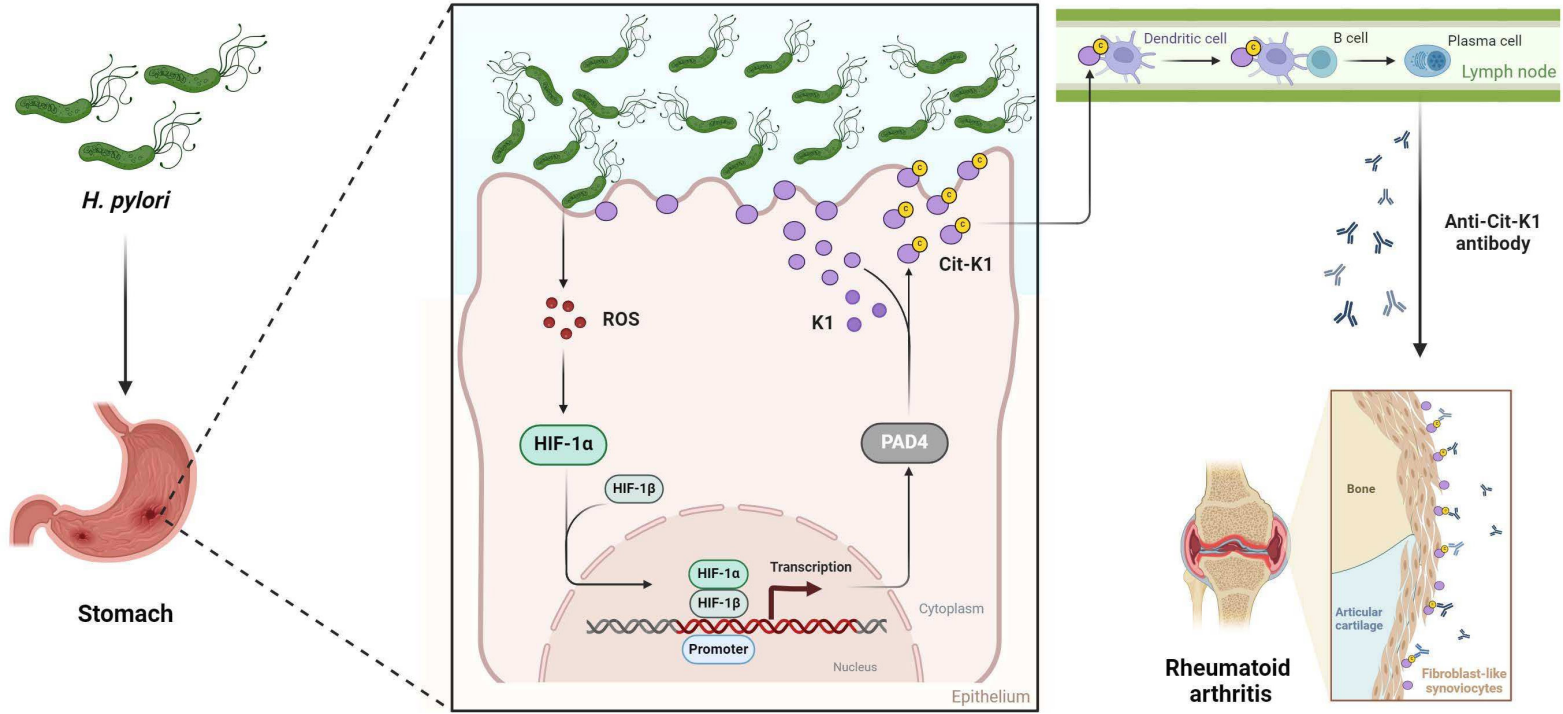
Schematic representation of the mechanism by which *H. pylori* infection exacerbates RA. Created with Biorender.com. HIF-1α, hypoxia-inducible factor 1-alpha; RA, rheumatoid arthritis; ROS, reactive oxygen species.

In conclusion, we propose a mechanism through which *H. pylori* infection may worsen RA (figure 8). We found that *H. pylori* infection upregulates PAD4 via the ROS/HIF-1α pathway. Moreover, highly expressed PAD4 could citrullinate K1, a cell membrane protein, the generated Cit-K1 may be picked up by dendritic cells either as cell debris or by other unknown mechanisms to eventually activate Cit-K1-specific B cells in the local lymph nodes to produce anti-Cit-K1 antibodies. Finally, the anti-Cit-K1 antibody-Cit-K1 immune complexes in the synovial space may initiate or further enhance an existing inflammatory response. Therefore, the categorisation of patients with RA based on their *H. pylori* infection status, and the implementation of standardised protocols for *H. pylori*-associated antibiotic therapy in infected patients with RA, could offer a significant therapeutic approach to better managing RA. Furthermore, such anti-Cit-K1 antibody exhibits potential as a dependable biomarker for prognosticating the severity of RA.

## supplementary material

10.1136/ard-2023-225306 online supplemental file 1

10.1136/ard-2023-225306 online supplemental file 2

10.1136/ard-2023-225306 online supplemental file 3

10.1136/ard-2023-225306 online supplemental file 4

10.1136/ard-2023-225306 online supplemental file 5

10.1136/ard-2023-225306 online supplemental file 6

10.1136/ard-2023-225306 online supplemental file 7

10.1136/ard-2023-225306 online supplemental file 8

10.1136/ard-2023-225306 online supplemental file 9

10.1136/ard-2023-225306 online supplemental file 10

10.1136/ard-2023-225306 online supplemental file 11

10.1136/ard-2023-225306 online supplemental file 12

10.1136/ard-2023-225306 online supplemental file 13

10.1136/ard-2023-225306 online supplemental file 14

10.1136/ard-2023-225306 online supplemental file 15

## Data Availability

Data are available on reasonable request.
