# Supplementary figures and images for: Helicobacter pylori upregulates PAD4 expression via stabilising HIF-1α to exacerbate rheumatoid arthritis
Source: Ann Rheum Dis. 2024 Aug 6;83(12):e225306. doi: 10.1136/ard-2023-225306 (PMC11671999; doi:10.1136/ard-2023-225306)

A

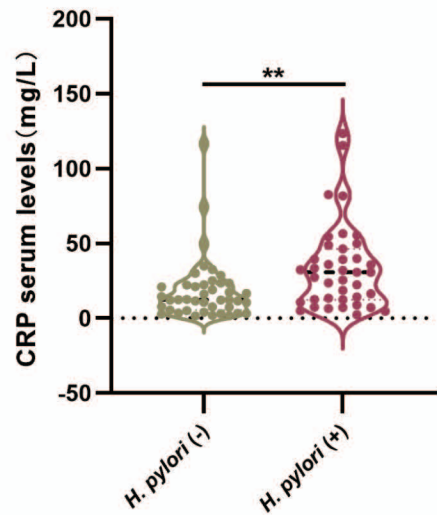

B

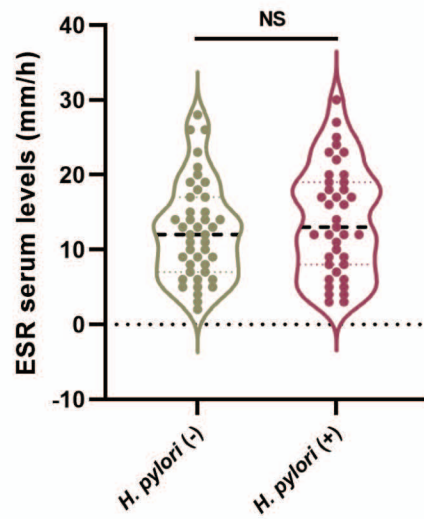

C

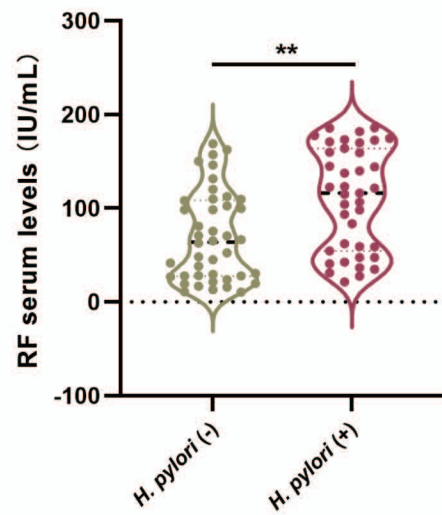

D

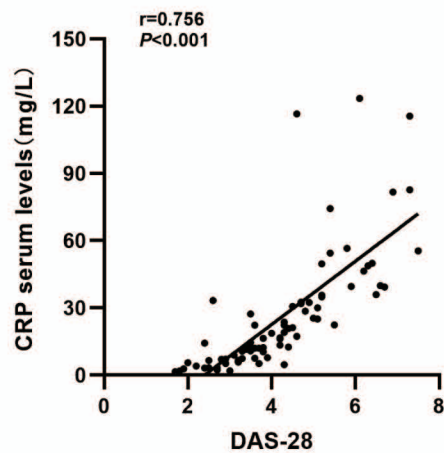

E

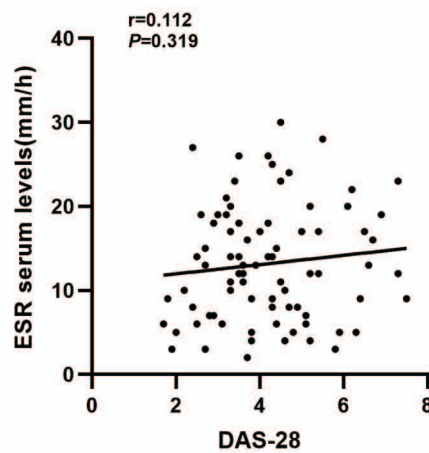

F

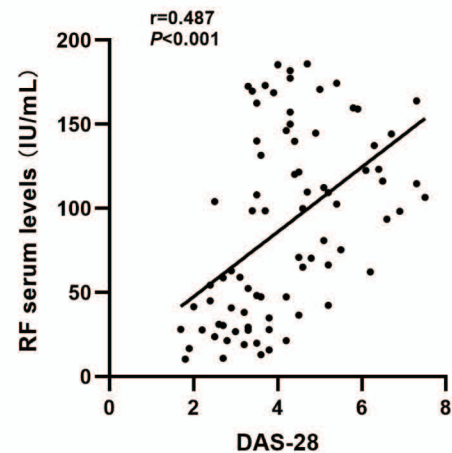

Supplement: online supplemental file 1 [file ard-83-12-s001.pdf]

**A**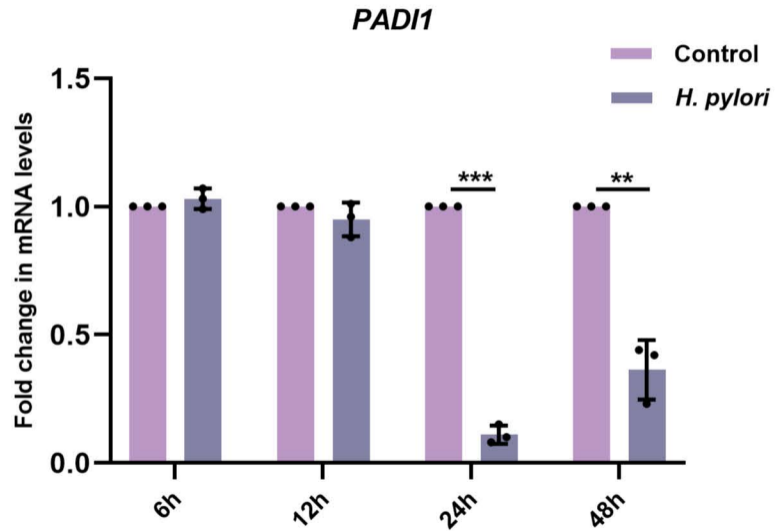**B**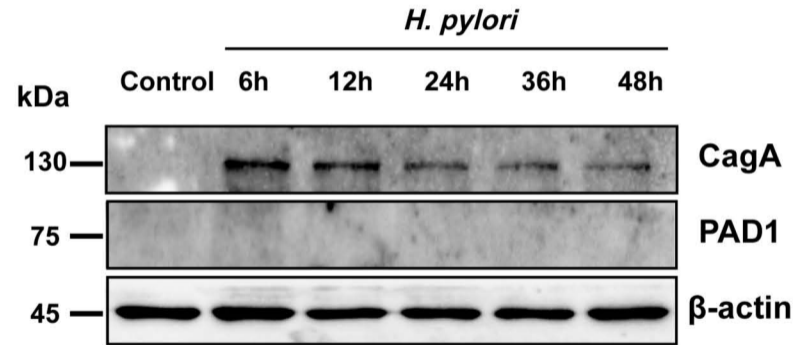

Supplement: online supplemental file 2 [file ard-83-12-s002.pdf]

**A**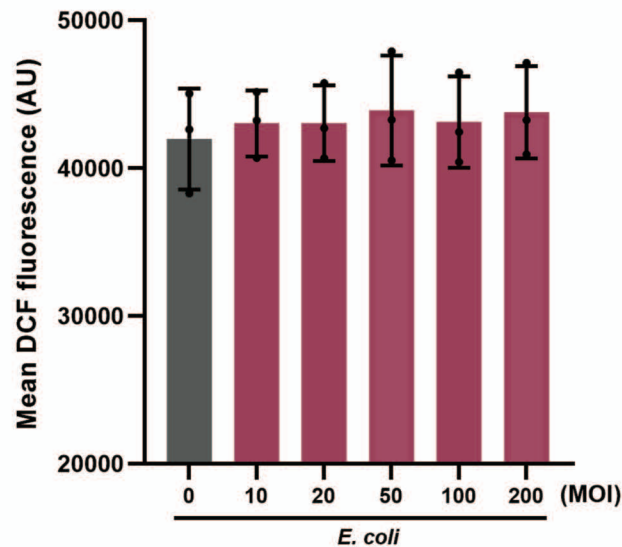**B**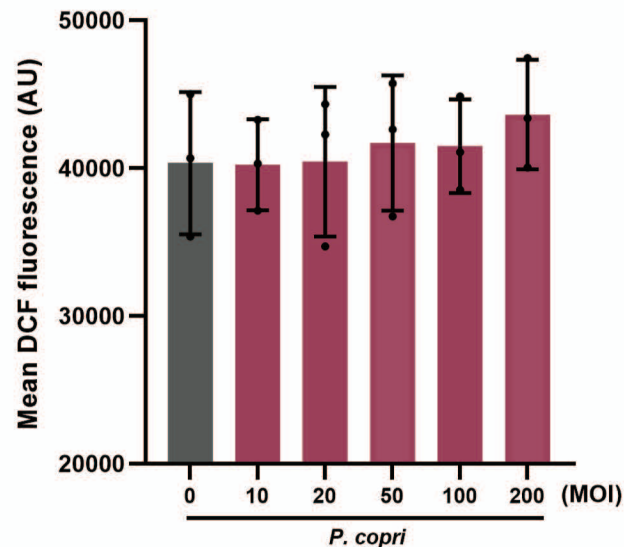**C**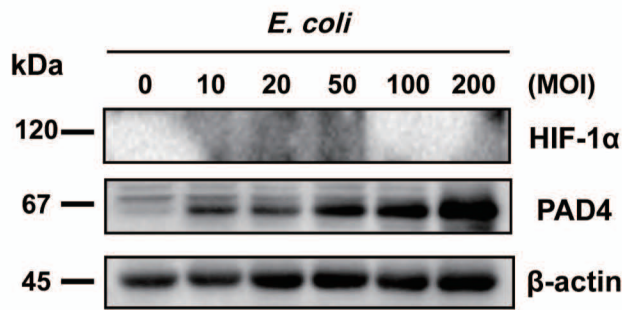**D**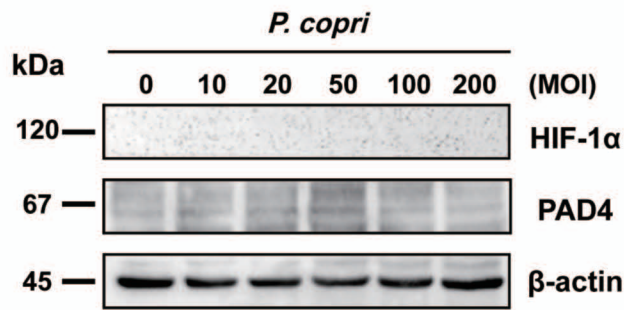

Supplement: online supplemental file 3 [file ard-83-12-s003.pdf]

**A**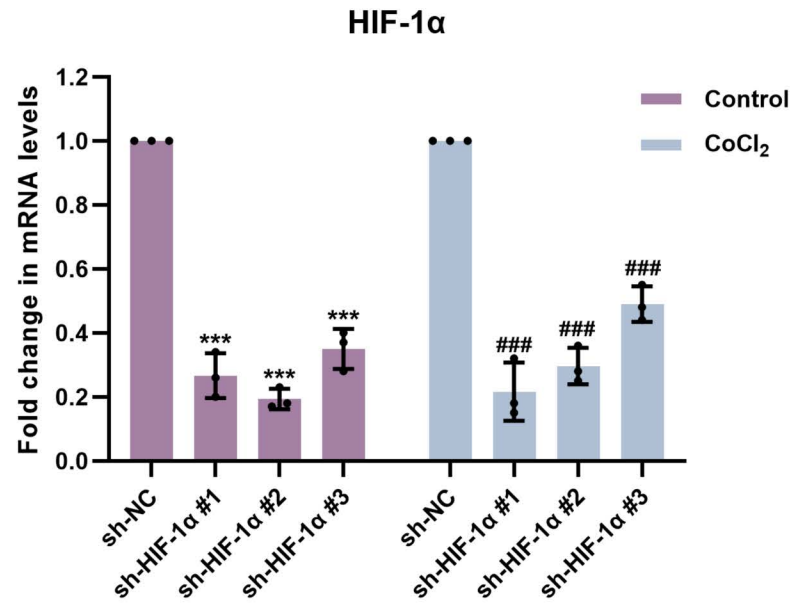**B**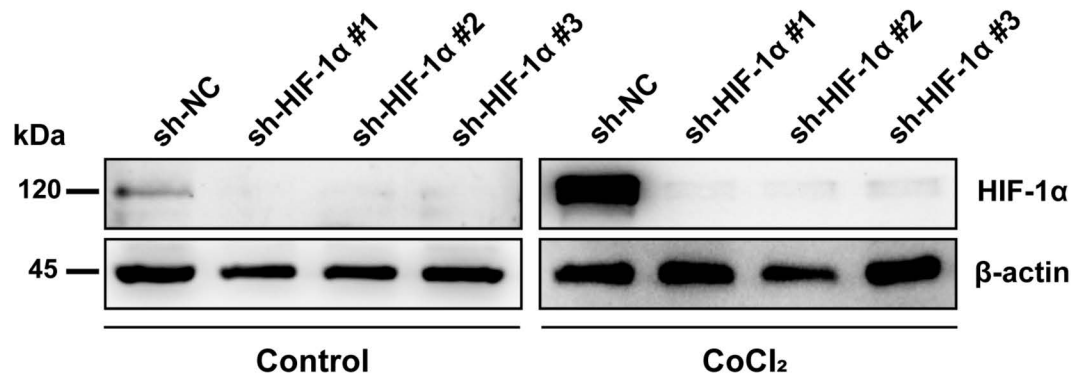

Supplement: online supplemental file 4 [file ard-83-12-s004.pdf]

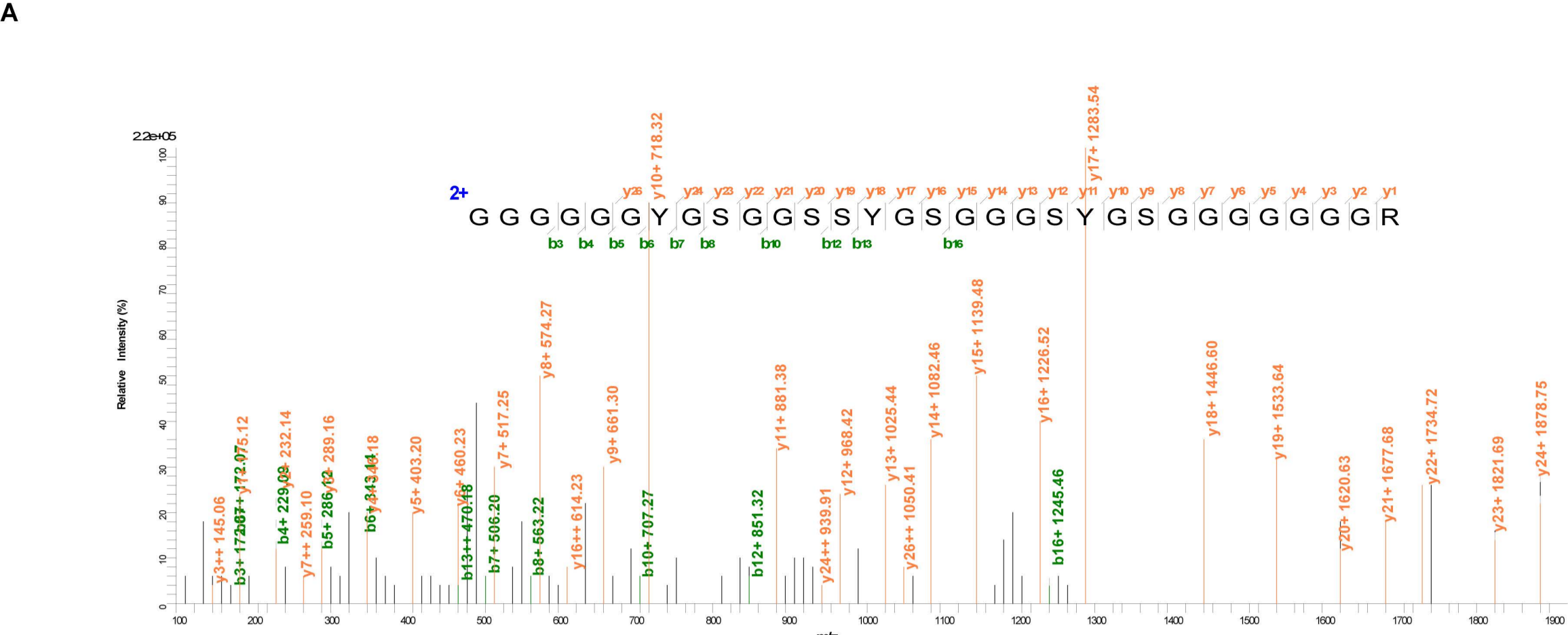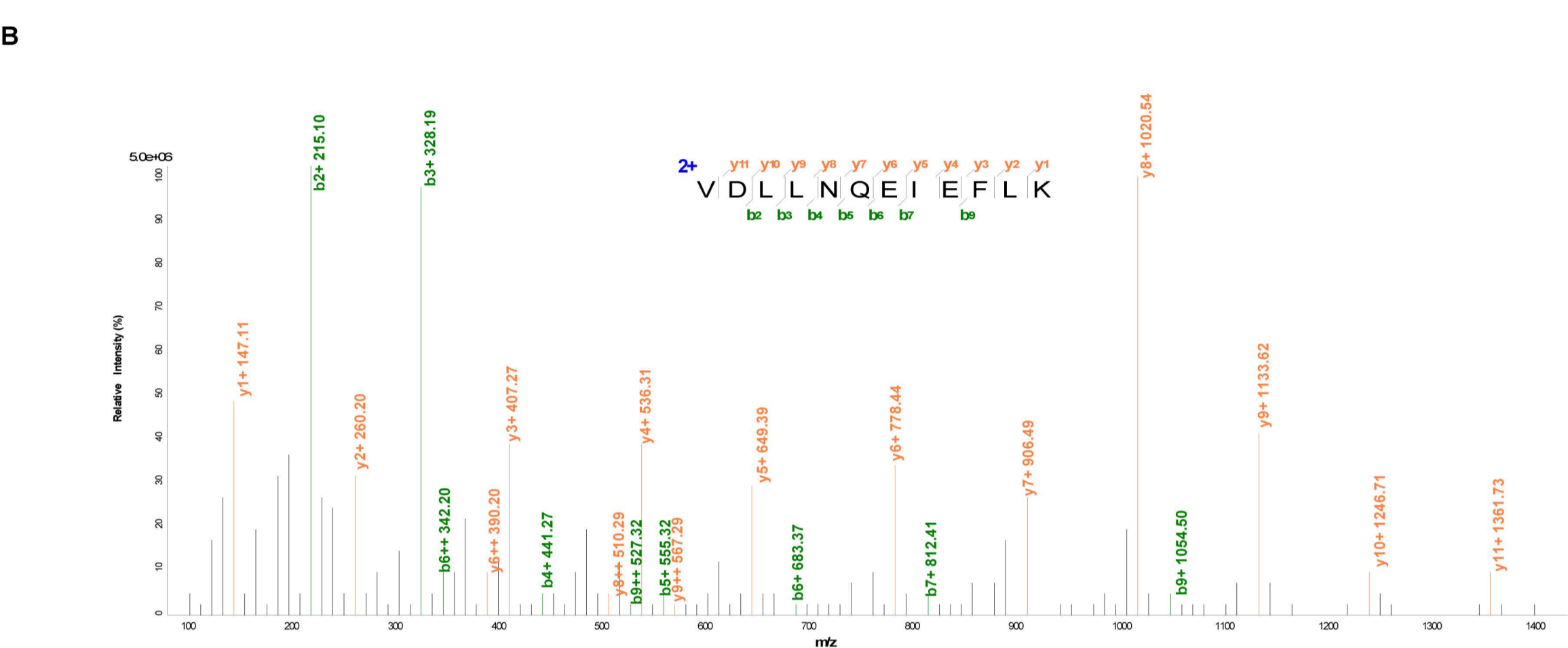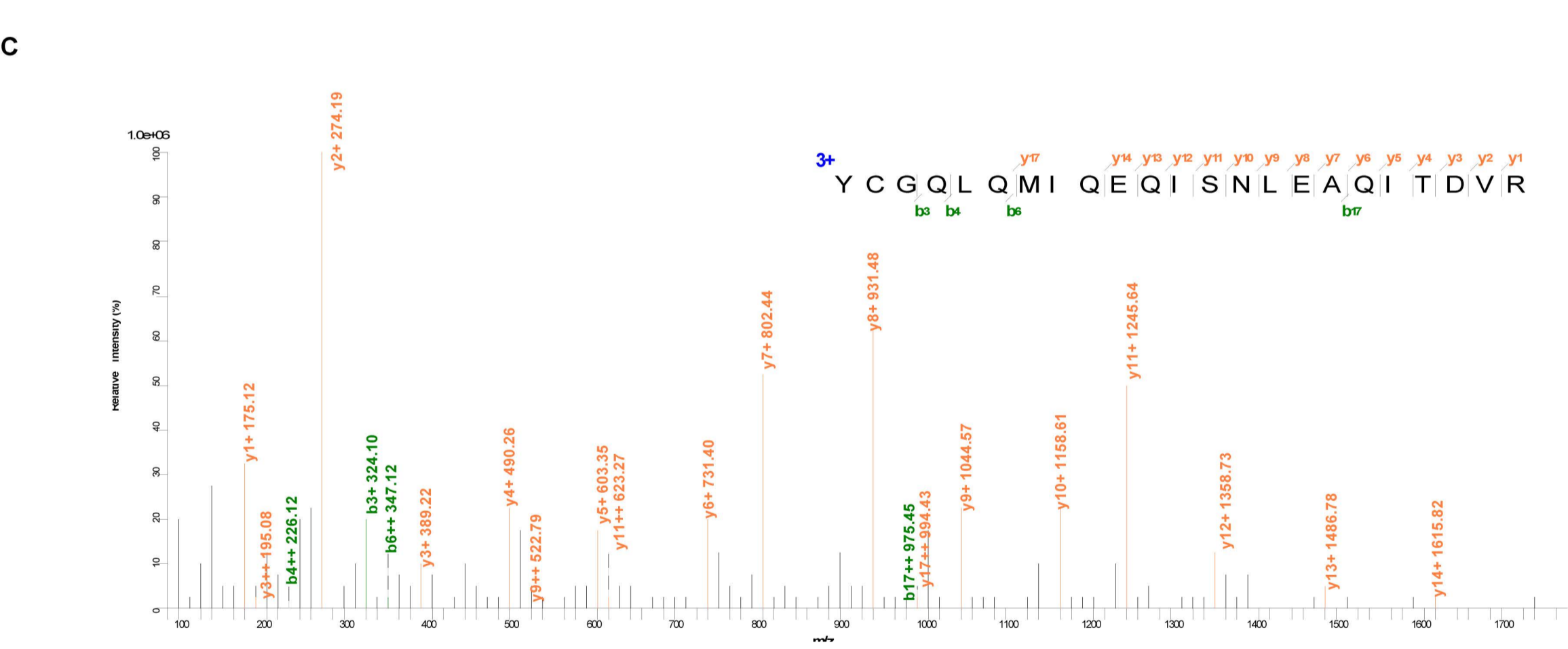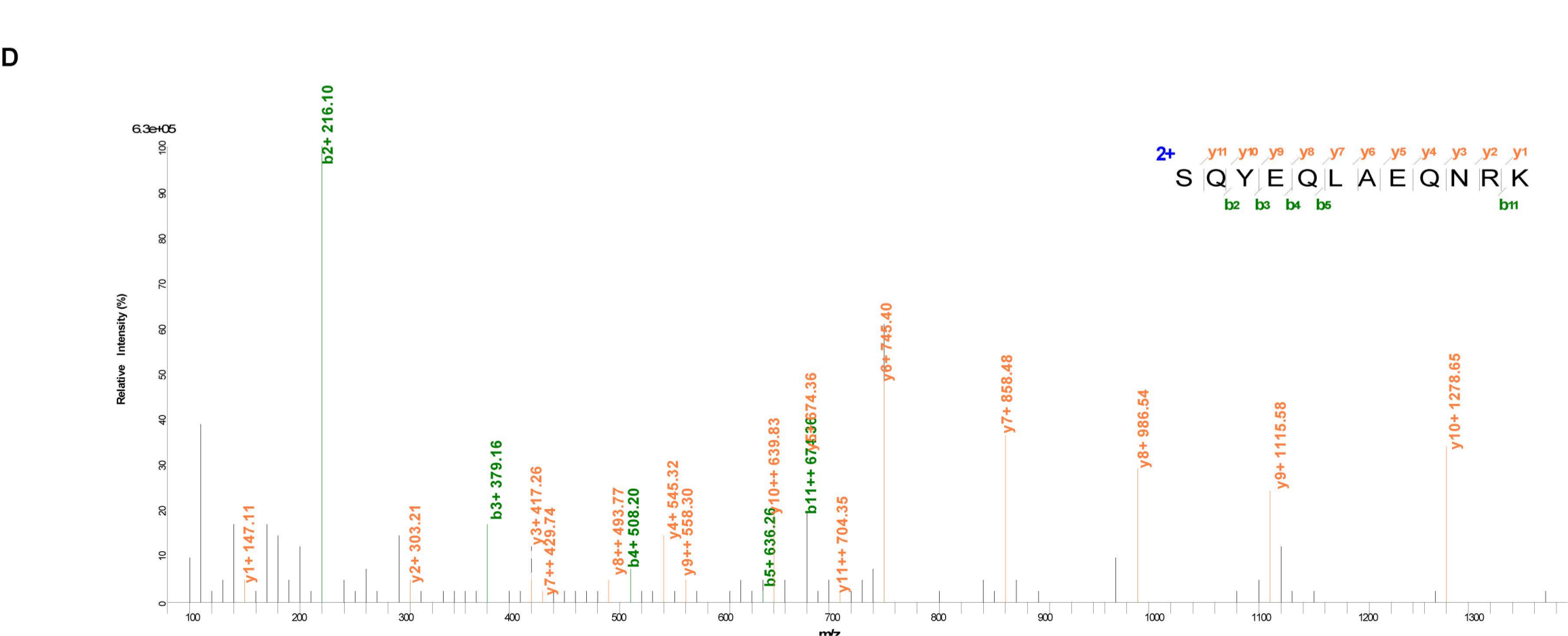

Supplement: online supplemental file 5 [file ard-83-12-s005.pdf]

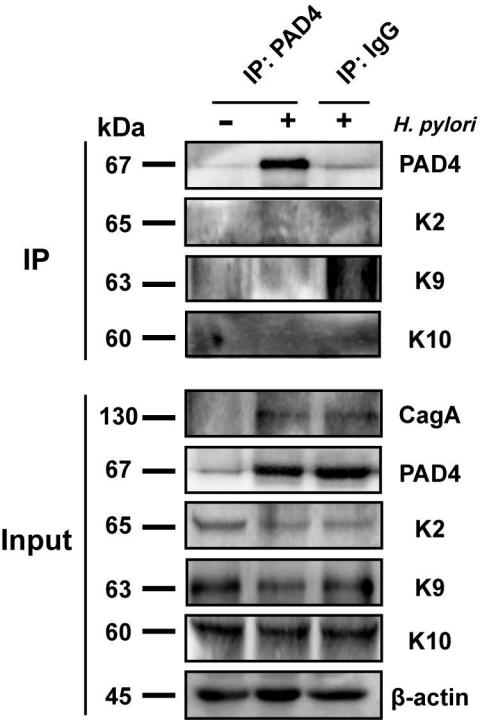

Supplement: online supplemental file 6 [file ard-83-12-s006.pdf]

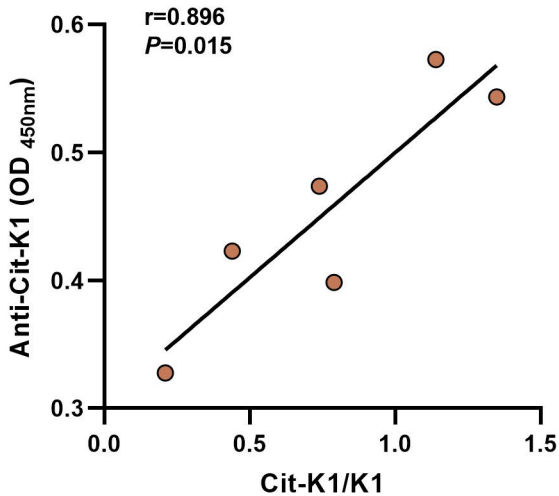

Supplement: online supplemental file 7 [file ard-83-12-s007.pdf]
