# Supplementary material for: Helicobacter pylori upregulates PAD4 expression via stabilising HIF-1α to exacerbate rheumatoid arthritis
Source: Ann Rheum Dis. 2024 Aug 6;83(12):e225306. doi: 10.1136/ard-2023-225306 (PMC11671999; doi:10.1136/ard-2023-225306)
Supplement: online supplemental file 8 [file ard-83-12-s008.pdf]

Methods

Subjects

Permission to perform the research was approved by the Eighth Affiliated Hospital of Sun Yat-sen University and Shenzhen Futian Hospital for rheumatic diseases. All subjects provided written informed consent. Serum samples were acquired from 81 patients with RA. Detailed patient information is summarized in Supplementary Table 1. Synovial fluids were obtained from RA patients undergoing arthrocentesis (n=7), and one synovial tissue was collected during joint replacement surgery (n=1). All patients were screened for eligibility at the outpatient clinic, with RA diagnosed according to the revised 2010 American College of Rheumatology (ACR) criteria.

H. pylori detection

H. pylori infection assay was performed using the *Helicobacter pylori* antibody typing detection kit (BLOT, Shenzhen, China). Briefly, 1 mL working buffer and 10 μL serum were placed in a tube containing a blotting membrane. The tube was incubated for 30 min on a rotary shaker. After discarding the liquid, the tube was washed three times. Subsequently, 1 mL buffer and 10 μL enzyme-linked reagent were added and incubated for 30 min. Finally, a chromogenic reagent was added for color development. The blotting membrane was compared to the standard belt picture to determine the presence or absence of *H. pylori* infection. We defined the *H. pylori* status as positive (*H. pylori* +) if cytotoxin-associated gene A (CagA), vacuolating cytotoxin (VacA), urease A (UreA), and urease B (UreB) were all positive and as negative (*H. pylori* -) if all of them were negative.

Cell culture and treatment

The normal human gastric epithelium cell line GES-1 (CL-0563) was purchased from Procell Life Science & Technology Co., Ltd (Wuhan, China). The human RA fibroblast-like synoviocyte MH7A cell line was obtained from the Riken cell bank (RCB1512, Ibaraki, Japan). Both GES-1 cells and MH7A cells were cultured in RPMI 1640 (C11875500BT, GIBCO, Thermo Fisher Scientific, Massachusetts, USA) with 10% fetal calf serum (FCS) (04-001-1A, Biological Industries, Kibbutz Beit Haemek, Israel) and 1% Penicillin-Streptomycin Solution (15140122, GIBCO, Thermo Fisher Scientific, Waltham, Massachusetts, USA). All cells were maintained at 37°C with 5% CO_2. In some experiments, GES-1 cells were treated for 48 h with 100 μM $CoCl_2$ (15862-1ML-F, Sigma-Aldrich, Burlington, Massachusetts, USA) to mimic hypoxic conditions.

Strains, cultural conditions, and infection

The wild-type *H. pylori* strain NCTC 11637, and *E. coli* strain BL21 were from the lab collection. The *P. copri* strain BNCC 337399 was purchased from the BeNa Culture Collection (Beijing, China). *H. pylori* was cultured on Trypticase soy agar containing 5% heat-inactivated newborn calf serum (HL1003-01, HALI Biotechnology, Chengdu, China) and 5% sheep’s blood (SHSJ0103, SHIGOYI, Nanjing, China) under

microaerophilic conditions (5% O_2, 10% CO_2, and 85% N_2) at 37°C for 48 h. *E. coli* was propagated in Luria-Bertani (LB) plates at 37°C for 12 h. *P. copri* was grown anaerobically on Columbia blood-agar plates (BNCC 352241, BeNa Culture Collection, Beijing, China) at 37°C for 48 h.

GES-1 cells were seeded in 6-well plates, grown to 80% confluency, and then infected with *H. pylori* for 6 h, 12 h, 24 h, 36 h, and 48 h or with *E. coli* and *P. copri* for 6 h.

Purification of human ACPA (anti-CCP IgG antibodies)

Total IgGs were purified from the serum of RA patients using the protein G affinity column as described before[(1)]. Subsequently, IgGs were diluted to 1:100 and transferred into the CCP plate (JL14854-96T, Jianglai, Shanghai, China). Antibodies recognizing CCP were eluted from the plate using elution buffer (0.1 M glycine, pH = 2.8) according to the literature method[(2)]. The eluates were pooled and dialyzed overnight in PBS at 4°C and stored at -80°C for further studies.

Cell counting kit-8 (CCK-8) assay

Cell proliferation was determined by using the CCK-8 assay (CK04, Dojindo Molecular Technologies, Kyushu, Japan). MH7A cells were cultured in 96-well plates (5×10^3 cells/well) for 24 h, followed by treatment with polyclonal ACPA at different times. Then, cells were incubated with CCK-8 reagent for another 2 h, and the absorbance was examined at 450 nm.

ELISA assay

The levels of PAD4 in serum were quantified by an ELISA kit purchased from Wuhan Huamei Biological Engineering Co., Ltd (CSB-E16219h, Wuhan, China). ACPA levels in synovial fluid were determined by the ELISA kit (JL14854-96T, Jianglai, Shanghai, China). The levels of IL-6 and IL-8 in the cell supernatant were measured using human IL-6 (EHC007.96, NeoBioscience, Shenzhen, China) and IL-8 (EHC008.96, NeoBioscience, Shenzhen, China) ELISA kits, respectively. All experiments were performed according to the product instructions.

PAD4 enzymatic activity

The antibody-based assay for PAD activity (ABAP) was carried out as previously described[(3)]. A 96-well plate was coated with a peptide (3.6 μg/ml) containing arginine in a coating buffer (abs9289, Absin, Shanghai, China) overnight at 4 °C. As a positive control, a citrulline-containing peptide was applied. Subsequently, 30 μg of protein lysates from GES-1 cells untreated or treated with *H. pylori* at a different time, in the presence or absence of 200 μM Cl-A (HY-100574A, MedChemexpress, Monmouth Junction, New Jersey, USA), were diluted with deimination buffer (40 mM Tris-HCl, 5 mM $CaCl_2$, 1 mM DTT, pH 7.5). All samples were added to the plate and incubated for 2 hours at 37 °C. To detect citrullinated peptides, Anti-Citrullinated Fibrinogen antibody (1:1000, MQR1.101, ImmunoPrecise Antibodies, Fargo, North Dakota, USA) was incubated overnight at 4 °C in a blocking solution (1% BSA and 0.05% Tween-20 in PBS). After washing five times with PBST (0.05% Tween-20 in PBS), the plate was

incubated with goat anti-mouse IgG HRP-conjugated secondary antibody (1:1000, SA00001-1, Proteintech, Rosemont, IL, USA) at 37 °C for 1h. Next, 100 µl TMB (PA107-01, TIANGEN, Beijing, China) was added to each well, and the reaction was stopped by 100 µl stop solution (E661006-0100, Sangon Biotech, Shanghai, China). The samples were measured at 450 nm using a microplate reader (TECAN Spark, Männedorf, Switzerland).

RNA extraction, reverse transcription, and quantitative nucleic acid analyses

Total RNA was isolated using the SteadyPure Universal RNA Kit II (AG21022, Accurate Biotechnology, Changsha, China), and 1 µg RNA was reverse transcribed into cDNA with HiScript III RT SuperMix for qPCR (R323-01, Vazyme, Nanjing, China). All primers were designed with Primer 3 and synthesized by Sangon Biotech (Supplementary Table 2). Quantitative real-time PCR (RT-qPCR) was performed using the Taq Pro Universal SYBR qPCR Master Mix (Q712-02, Vazyme, Nanjing, China) on a LightCycler 480 Instrument II (Roche, Basel, Switzerland). The relative transcript expression levels of samples were normalized to the β-actin according to the cycle threshold ($2^{-\Delta\Delta CT}$) method.

Immunoblotting analysis

Cultured cells were lysed in RIPA buffer (P0013B, Beyotime, Shanghai, China) supplemented with a protease inhibitor cocktail (K1012, APExBIO, Houston, USA). After examining protein concentrations with a BCA assay kit (P0010, Beyotime, Shanghai, China), equal amounts of protein were separated by SDS-PAGE and transferred to Polyvinylidene fluoride (PVDF) membranes (IPVH00010, Merck Millipore, Burlington, Massachusetts, USA). Membranes were blocked for 2 h in 5% skim milk and then incubated with a primary antibody (Supplementary Table 3) overnight at 4 °C. Subsequently, membranes were washed with TBST (0.1% Tween-20 in TBS) and incubated with an HRP-conjugated secondary antibody for 2 h at room temperature. Immunoreactive proteins were visualized using SuperSignal™ West Pico PLUS (34577, Thermo Fisher Scientific, Waltham, Massachusetts, USA) on a ChemiDoc™ Touch machine (Bio-Rad, Hercules, California, USA).

Luciferase assay

A 2000 bp wild-type or mutant *PADI4* promoter sequence was synthesized and inserted into the GV238 plasmid (Genechem, Shanghai, China; Supplementary Table 4). The empty GV238-basic plasmid was used as the control plasmid. GES-1 cells (3×10^4/well) were co-transfected with 450 ng luciferase reporter plasmid and 50 ng TK promoter-Renilla-Luciferase plasmid using Lipofectamine™ 3000 (L3000001, Thermo Fisher Scientific, Waltham, Massachusetts, USA). After 24 h, cells were infected with *H. pylori* (MOI=100) for 12 h or treated with 100 µM $CoCl_2$ for 48 h. The luciferase activities (Firefly and Renilla luciferase) were assayed with a Dual-Luciferase assay kit according to the manufacturer's instructions (DD1205, Vazyme, Nanjing, China). The results were expressed as the relative firefly luciferase activity ratio to Renilla luciferase activity.

Lentiviral transduction

Lentiviral vectors encoding shRNA targeting HIF-1α were purchased from Genechem (Shanghai, China; Supplementary Table 5). GES-1 cells were seeded into 12-well plates (4×10^4 cells/well). Subsequently, cells were transfected with a multiplicity of infection (MOI) of 20, and 20 µl HiTransG-P (Genechem, Shanghai, China) was added to promote efficiency. After 24 h, transduced cells were selected with 1.5 µg/ml puromycin (B7587, APExBIO, Houston, Texas, USA) for 7 days, followed by 0.75 µg/ml puromycin maintenance.

Protein pull-down and immunoprecipitation

Uninfected and *H. pylori*-infected GES-1 cells were washed with PBS and lysed in cell lysis buffer for Western and IP (P0013, Beyotime, Shanghai, China) containing protease inhibitors. Then, 1 mg of lysates were pre-cleared with 20 µL Protein A/G PLUS-Agarose (sc-2003, Santa Cruz Biotechnology, Dallas, Texas, USA) for 4 h with rotation. After centrifugation (3000 rpm, 3 min), 50 µg of supernatant was taken as input. Next, the remaining supernatant was incubated on a rotary wheel with 4 µg anti-PAD4 antibody (17373-1-AP, Proteintech, Rosemont, IL, USA) or isotype control and 20 µL Protein A/G PLUS-Agarose overnight at 4 °C. The immune complexes were collected by centrifugation at 3000 rpm, washed three times with PBS, resuspended in a loading buffer (P0015L, Beyotime, Shanghai, China), and boiled for 10 min. Finally, the interacting proteins of PAD4 were analyzed by mass spectrometry and identified by immunoblotting.

Mass spectrometry

Mass spectrometry was performed at Applied Protein Technology Co., Ltd (Shanghai, China). Initially, pull-down samples were run on a 10% SDS-PAGE gel, and a gel band was cut and digested with trypsin at 37 °C overnight. The peptide mixture from each sample was separated using a C18-reversed phase analytical column. LC-MS/MS analysis was conducted on a Q Exactive Mass Spectrometer (Thermo Fisher Scientific, Waltham, Massachusetts, USA) coupled to an Easy nLC (Thermo Fisher Scientific, Waltham, Massachusetts, USA). For peptide and protein identification, MS/MS fragmentation spectra were searched for protein identification using the Mascot engine (Matrix Science, London, UK; version 2.2) against a non-redundant International Protein Index Arabidopsis sequence database v3.85 from the European Bioinformatics Institute (http://www.ebi.ac.uk/). Peptide and MS/MS tolerances were set to 20 parts per million (ppm) and 0.1 daltons (Da). Trypsin was set as an enzyme and allowed up to 2 missed cleavages. Oxidation (M) was considered variable, whereas Carbamidomethylation (C) was considered fixed modification.

ROS measurement

Intracellular ROS levels were determined using a ROS Fluorometric Assay Kit (E-BC-K138-F, Elabscience, Wuhan, China). Before treatment with *H. pylori* for 6 h, GES-1 cells were incubated with Diphenyleneiodonium chloride (DPI; HY-100965,

MedChemexpress, Monmouth Junction, New Jersey, USA) or N-Acetylcysteine (NAC; HY-B0215, MedChemexpress, Monmouth Junction, New Jersey, USA) for one hour. The supernatant was discarded, and the cells were washed in RPMI 1640 medium. 10 mM DCFH-DA was added to each plate for 60 min at 37 °C. Digested cells were resuspended in RPMI 1640 medium, and DCF fluorescence was excited at 488 nm and detected at 525 nm.

Chromatin immunoprecipitation assays

Chromatin immunoprecipitation (ChIP) assays were carried out using the SimpleChIP® Enzymatic Chromatin IP Kit (9003S, Cell Signaling Technology, Danvers, Massachusetts, USA). Initially, GES-1 cells were treated with $CoCl_2$ for 48 h, fixed with 1% formaldehyde (final concentration) for 15 minutes, and stopped with glycine for 5 minutes. Immediately after that, chromatin was fragmented into 150-900 bp by micrococcal nuclease and sonication. Specific DNA fragments were immunoprecipitated using an antibody directed against the DNA-binding protein of interest and ChIP-Grade Protein G Magnetic Beads. Subsequently, immunoprecipitated chromatin was washed with different gradients of elution buffer and purified by DNA purification columns. Finally, purified DNA was used for ChIP-qPCR analysis. Primer sequences for qPCR are provided in Supplementary Table 6.

In vitro citrullination of K1

Recombinant K1 purified from *E. coli* was custom-made by Abmart (EHH2503L, Shanghai, China). 1μg K1 was incubated with purified recombinant PAD4 (10500, Cayman Chemical Company, Ann Arbor, Michigan, USA) in 50 mM HEPES, containing 10 mM calcium chloride and 5 mM DTT, at 37°C for 4 h. Citrullination of K1 was detected using an anti-modified citrulline antibody (MABN328, Sigma-Aldrich, Burlington, Massachusetts, USA).

Immunoprecipitation of Cit-K1

Cit-K1 was immunoprecipitated from serum samples and synovial tissue. Firstly, samples were lysed in cell lysis buffer for Western and IP (P0013, Beyotime, Shanghai, China) and clarified by centrifugation. Subsequently, immunoprecipitated with 10 µg anti-K1 polyclonal antibody (16848-1-AP, Proteintech, Rosemont, IL, USA). Cit-K1 was analyzed by western blots using anti-K1 antibody (16848-1-AP, Proteintech, Rosemont, IL, USA) and anti-modified citrulline monoclonal antibody (MABN328, Sigma-Aldrich, Burlington, Massachusetts, USA).

Anti-citrullinated-K1 antibody assessment

Two methods were used to detect serum anti-Cit-K1 antibody levels[(4)]. Cit-K1 protein (4 µg/ml) was coated on a plate and incubated overnight at 4 °C. After washing, the plate was incubated with a blocking buffer (2% BSA in PBST) for 2 h. Serum samples (diluted 1:50) were added and incubated at 4°C for 24 h, followed by goat anti-human IgG HRP-conjugated secondary antibody (1:2000) for 2 h and then by TMB for 5 min. The reaction was stopped by adding a stop solution, and the plate was immediately

read at 450nm. Control experiments were performed by coating irrelevant protein (5% BSA) on the plate, and the rest of the procedures were the same as described above. To analyze levels of anti-Cit-K1 antibody, we defined the mean plus three standard deviations of OD_{450} values from healthy controls as the cut-off (OD_{450}=0.45). A serum sample with an OD value ≥ 0.45 was considered anti-Cit-K1 antibody positive. The synovial fluid samples from RA patients were diluted to 1:50, and the anti-Cit-K1 antibody was analyzed with ELISA according to the above instructions.

Cit-K1 protein (1 µg) was subjected to SDS-PAGE and transferred to a PVDF membrane. The blot was incubated overnight with a serum that was positive for anti-Cit-K1 antibody (1:50) at 4°C and a goat anti-human IgG secondary antibody (1:5000) for 2 h at room temperature. Protein bands were visualized with the ChemiDoc™ Touch machine (Bio-Rad, Hercules, California, USA).

GO analysis

The raw data were downloaded from the public GEO database (http://www.ncbi.nih.gov/geo). The software package Limma was used to identify differentially expressed mRNA. The R package ClusterProfiler (version: 3.18.0) was employed to analyze the GO function of upregulated genes (Supplementary Table 7).

Statistical analysis

All statistical analyses were performed utilizing SPSS version 23.0 for Windows (SPSS Software, Chicago, USA). The Chi-square test compares gender, age, and treatment between *H. pylori*-negative and *H. pylori*-positive RA patients. Data were expressed as the mean ± standard deviation (SD). All the cell-based, in vitro experiments were independently repeated three times. An independent sample Student's t-test or Mann-Whitney U test was utilized to evaluate the differences between the two groups. Multigroup comparisons were performed using a one-way or two-way analysis of variance (ANOVA). One-way ANOVA is followed by Bonferroni's or Tamhane's T2 test, while for two-way ANOVA, post hoc analysis was performed using the LSD test. Correlations were assessed with the Pearson coefficient test. P values less than 0.05 were considered significant.

Supplementary figure legend

Supplementary Figure 1

Effect of *H. pylori* infection on CRP, ESR, and RF in RA patients. (A-C) Serum levels of (A) CRP, (B) ESR, and (C) RF in *H. pylori*-negative (n = 42) and *H. pylori*-positive (n=39) RA patients. (D-F) Correlation between DAS-28 and (D) CRP, (E) ESR, (F) RF. Data are presented as violin plots with the median (black dotted line) and quartiles (green and red dotted lines) indicated. Statistical analyses were performed using the Mann–Whitney U test (A, C) and Student's t-test (B). **P<0.01. RA, rheumatoid arthritis; DAS-28, disease activity score 28; CRP, C-reactive protein; ESR, erythrocyte sedimentation rate; RF, rheumatoid factor.

Supplementary Figure 2

PAD1 expression during *H. pylori* infection. (A) RT-qPCR analysis of *PADI1* mRNA expression levels in *H. pylori*-infected GES-1 cells (MOI=100). (B) Western blot analysis of PAD1 in GES-1 cells infected with *H. pylori* (MOI=100) for different periods. Data are presented as mean ± SD. Statistical analyses were performed with Student's t-test. **P<0.01, ***P<0.001. PAD1, *PADI1*, Protein arginine deiminase type 1; CagA, cytotoxin-associated gene A; MOI, multiplicities of infection.

Supplementary Figure 3

Effect of *E. coli* and *P.copri* infection on ROS/HIF-1α pathway. (A-B) Detection of ROS levels in GES-1 cells infected with (A) *E. coli* or (B) *P. copri* (MOI=0, 10, 20, 50, 100, and 200) for 6 h. (C-D) GES-1 cells were treated with (C) *E. coli* or (D) *P. copri* at different MOI for 6 h. The expression of HIF-1α and PAD4 were determined by western blot. Data are presented as mean ± SD. Statistical analyses were performed with the one-way ANOVA. HIF-1α, hypoxia-inducible factor 1-alpha; PAD4, protein arginine deiminase type 4; ROS, reactive oxygen species; *E. coli*, Escherichia coli; *P. copri*, Prevotella copri; MOI, multiplicities of infection; ANOVA, analysis of variance.

Supplementary Figure 4

Validation of HIF-1α shRNA knockdown efficiency. (A) RT-qPCR analysis of HIF-1α mRNA level and (B) western blot analysis of HIF-1α protein level in GES-1 cells transfected with lentivirus-sh HIF-1α after treatment with or without $CoCl_2$ (100 µM, 48 h). Data are presented as mean ± SD. Statistical analyses were performed with a one-way ANOVA. ***P<0.001 vs control+sh-NC group; ###P<0.001 vs $CoCl_2$+sh-NC group. HIF-1α, hypoxia-inducible factor 1-alpha; $CoCl_2$, cobalt chloride; ANOVA, analysis of variance.

Supplementary Figure 5

MS spectrum of (A) K1, (B) K2, (C) K9, and (D) K10. MS, mass spectrometry; K1, Keratin 1; K2, Keratin 2; K9, Keratin 9; K10, Keratin 10.

Supplementary Figure 6

K2, K9, and K10 did not bind to PAD4. GES-1 cells were infected with *H. pylori* (MOI=100) for 6 h. PAD4 was immunoprecipitated (IP), and eluates were analyzed for K2, K9, and K10 by western blot. PAD4, protein arginine deiminase type 4; K2, keratin 2; K9, keratin 9; K10, keratin 10.

Supplementary Figure 7

Correlation analysis between Cit-K1 and anti-Cit-K1 antibody in the sera of RA patients (n=6). Cit-K1, citrullinated Keratin 1; K1, Keratin 1.

References

1. Ossipova E, Cerqueira CF, Reed E, et al. Affinity purified anti-citrullinated protein/peptide antibodies target antigens expressed in the rheumatoid joint. Arthritis Res Ther. 2014;16(4):R167.
2. Ioan-Facsinay A, el-Bannoudi H, Scherer HU, et al. Anti-cyclic citrullinated peptide antibodies are a collection of anti-citrullinated protein antibodies and contain overlapping and non-overlapping reactivities. Annals of the rheumatic diseases. 2011;70(1):188-93.
3. Zendman AJ, Raijmakers R, Nijenhuis S, et al. ABAP: antibody-based assay for peptidylarginine deiminase activity. Anal Biochem. 2007;369(2):232-40.
4. Li K, Mo W, Wu L, et al. Novel autoantibodies identified in ACPA-negative rheumatoid arthritis. Annals of the rheumatic diseases. 2021;80(6):739-47.
