# Supplementary material for: Helicobacter pylori upregulates PAD4 expression via stabilising HIF-1α to exacerbate rheumatoid arthritis
Source: Ann Rheum Dis. 2024 Aug 6;83(12):e225306. doi: 10.1136/ard-2023-225306 (PMC11671999; doi:10.1136/ard-2023-225306)
Supplement: online supplemental file 9 [file ard-83-12-s009.pdf]

Supplementary Table 1 Demographic characteristics and clinical data of RA

	H. pylori (-) RA	*H. pylori* (+) RA	P value
**N**	42	39	
**Sex, female n (%)**	33 (78.6%)	29 (74.4%)	0.655
**Age group n (%)**			0.959
21-30 years	4 (9.5%)	3 (7.7%)	
31-40 years	5 (11.9%)	4 (10.3%)	
41-50 years	8 (19.0%)	6 (15.4%)	
51-60 years	14 (33.3%)	13 (33.3%)	
61-70 years	11 (26.2%)	13 (33.3%)	
**Clinical data (mean ± SD)**			
DAS-28	3.68±1.04	4.73±1.53	0.001
ACPA	194.04±92.66	248.44±100.46	0.006
CRP	18.42±20.90	33.61±28.49	0.002
ESR	12.48±6.52	13.95±7.27	0.339
RF	69.40±47.87	109.97±54.69	0.001
**Treatment n (%)**			0.496
Methotrexate	10 (23.8%)	7 (17.9%)	
Leflunomide	8 (19.0%)	9 (23.1%)	
Hydroxychloroquine	12 (28.6%)	9 (23.1%)	
Etanercept	6 (14.3%)	11 (28.2%)	
Tocilizumab	6 (14.3%)	3 (7.7%)	

RA, rheumatoid arthritis; DAS-28, disease activity score 28; ACPA, anti-citrullinated protein antibody; CPR, C-reactive protein; ESR, erythrocyte sedimentation rate; RF, rheumatoid factors; *H. pylori*, *Helicobacter pylori*.
