# Supplementary material for: Helicobacter pylori upregulates PAD4 expression via stabilising HIF-1α to exacerbate rheumatoid arthritis
Source: Ann Rheum Dis. 2024 Aug 6;83(12):e225306. doi: 10.1136/ard-2023-225306 (PMC11671999; doi:10.1136/ard-2023-225306)
Supplement: online supplemental file 10 [file ard-83-12-s010.pdf]

**Supplementary Table 2 The primer sequences for RT-qPCR**

| | Gene name | Forward (5'-3') | Reverse (5'-3') |
|---|---|---|---|
| 1 | **β-actin** | CCTGGCACCCAGCACAAT | GGGCCGGACTCGTCATAC |
| 2 | **HIF-1α** | TAGCCGAGGAAGAACTATGAAC | CTGAGGTTGGTTACTGTTGGTA |
| 3 | ***PADI1*** | TCCAGAGACCCTGAAGCTGT | GTGCAGCTGTCCCTGAAGAT |
| 4 | ***PADI2*** | ACCTCCTCAGCCTCCCC | CCTACCTCTGGACCGATGTC |
| 5 | ***PADI3*** | GCGTCCCATAGACCTCAAAC | CAGAGAATCGTGCGTGTGTC |
| 6 | ***PADI4*** | CCTGTGGATTTCTTCTTGGC | GGGCACCTTGACTCAGCTT |
| 7 | ***PADI6*** | CAAGGTATAGGCGTGCTGGT | TCCTCCATACCTCCAAGGAA |
