# Supplementary material for: Helicobacter pylori upregulates PAD4 expression via stabilising HIF-1α to exacerbate rheumatoid arthritis
Source: Ann Rheum Dis. 2024 Aug 6;83(12):e225306. doi: 10.1136/ard-2023-225306 (PMC11671999; doi:10.1136/ard-2023-225306)
Supplement: online supplemental file 11 [file ard-83-12-s011.pdf]

Supplementary Table 3 Antibodies list

	Antibody	Application	Cat number	Source
**1**	Anti-modified citrulline, clone F95 Antibody	1:500 for WB	MABN328	Sigma-Aldrich, Burlington, Massachusetts USA
**2**	Anti-β-actin	1:1000 for WB	AF7018	Affinity Biosciences, Cincinnati, Ohio, USA
**3**	Anti-PAD1	1:1000 for WB	ab181762	Abcam, Cambridge, UK
**4**	Anti-PAD2	1:1000 for WB	12110-1-AP	Proteintech, Rosemont, IL,USA
**5**	Anti-PAD3	1:1000 for WB	ab172959	Abcam, Cambridge, UK
**6**	Anti-PAD4	1:1000 for WB or 4 μg for IP	17373-1-AP	Proteintech, Rosemont, IL,USA
**7**	Anti-HIF-1α	1:1000 for WB or 2 μg for ChIP	36169S	CST, Danvers, Massachusetts, USA
**8**	Anti-K1	1:1000 for WB or 10 μg for IP	16848-1-AP	Proteintech, Rosemont, IL,USA
**9**	Anti-K2	1:200 for WB	21725-1-AP	Proteintech, Rosemont, IL,USA
**10**	Anti-K9	1:500 for WB	ab171966-4	Abcam, Cambridge, UK
**11**	Anti-K10	1:500 for WB	18343-1-AP	Proteintech, Rosemont, IL,USA
**12**	Anti-CagA	1:200 for WB	sc-28368	Santa Cruz Biotechnology, Dallas, Texas, USA
**13**	Anti-CK1	1:1000 for WB	ab194344	Abcam, Cambridge, UK
**14**	Normal rabbit IgG	4 μg for IP	30000-0-AP	Proteintech, Rosemont, IL,USA
**15**	Anti-Citrullinated Fibrinogen antibody	1:1000 for ELISA	MQR1.101	ImmunoPrecise Antibodies, Fargo, North Dakota, USA
**16**	Goat anti-rabbit IgG, HRP-linked	1:3000 for WB	SA00001-2	Proteintech, Rosemont, IL,USA
**17**	Mouse anti-rabbit IgG (Light chain specific) HRP-linked	1:1000 for WB	SA00001-7L	Proteintech, Rosemont, IL,USA
**18**	Goat anti-mouse IgG, HRP-linked	1:1000 for WB	SA00001-1	Proteintech, Rosemont, IL,USA
**19**	Goat anti-human IgG, HRP-linked	1:5000 for WB or 1:2000 for ELISA	SA00001-17	Proteintech, Rosemont, IL,USA
**20**	Goat anti-mouse IgM (μ chain specific) HRP-linked	1:1000 for WB	F030210	Baiaolaibo Technology Co., Ltd., Beijing, China
