# Supplementary material for: Helicobacter pylori upregulates PAD4 expression via stabilising HIF-1α to exacerbate rheumatoid arthritis
Source: Ann Rheum Dis. 2024 Aug 6;83(12):e225306. doi: 10.1136/ard-2023-225306 (PMC11671999; doi:10.1136/ard-2023-225306)
Supplement: online supplemental file 12 [file ard-83-12-s012.pdf]

Supplementary Table 4 Wild-type (WT) and mutant (Mut) sequences that were inserted into firefly luciferase reporter plasmid

	Plasmid	Sequence
1	**GV238-*PADI4*-WT**	CAACAAAAACAAACAAACAAAAAGGCTCTATGAAGGCAGGATTTTTGTCCACTTTGTTCACAGCGTATCCACGGTGCCTGGAACAGTGCCTGGCCATCAATAGGCACTCAGTCAGTATTTGTTGAATTAACAAAACT<br>GCTTTGGGGCTCGTCCTCTTTGTGGCTCGTTCACAGCAAATTTAAACCCCAAACTGCTGAAGAAAATAAGAAAGAAAAGAAGGAACAGAAGAAAACAACCTCAAGAGACCTAAGACTTGGGGTAGAGGCCAGCCC<br>TCCCTCTGATTTGCTGTGTGACCGTGGGCAGGTCCCACTCCCTTTCTGTGCTTCAGTCTCCCCTTCTGCAAAACGAGGACTGGATTGGGTGTTCTCTAAGGGCCCTCCCAACTCTAACAATGGACAGCTCCCTCC<br>TGCCACTTCCCAGTAGAAGCTCAAACTAGTTCTAATCATGCGGTCAGCCAGAGAAATTGGGTTTATTAACACTGTGTGCAGGCAGTCATGTAGATGCGTCAGAACGTCGGACCTGGCCACATCATCATCTGCTGGTA<br>CAGAAGATAAATTTTGAGCCCAGAGAAGGTGGTCTGAATCCAGATCCCCACTGTAGGGCTTATGAGACCTTGGGGGTCTCACTCAATCTCTCTAAGTCTTTGTGGTCTCATCTACACATGGGCATCCTGATAGGACG<br>CACTCCATAGGGATGTTGAGATGAGGCAGGATTTGAACTAAAGCCTGTTTGATCCCACAGCCTGACTCTGAATTAATGAGGCAAATTCTTTGCTTCAAAGCCAAAGCAAGCAAACAAAAAACAAACGCAAGATATCC<br>AACCCACGAAACGCTCTATGTTCACGCCAGACTCCCATGCATGCACCCTTTGAGTGCTCCTTGTTGGGACCCATCCACCACCCCCCAGTTGCCTCATTAAAGGCAGAGCCTGGCACCAATGGCCCAGGTGCAAC<br>CACAGCTCTGAGGCCACATGGGCATCCCCCTGGCAGGCGTGGCCCACACCTGCACTGTCTGGTCTGACACCCAGAGGCCCTGGCAAGAGGCAGGTATCCTGGAGCATGCAGAGAACATCAACTTCCATGCCAG<br>GAAGCTCATCTTCTCTTCCCAGCTCTGCCGCATTCTAGCTCTGGCACCTTGGGCAAGAGCTAGTCACTTTTACTCTCTGAGCCTCAATTTTCCTTCTGTAAAATGGGTTGTAATAATAAGCCCATATACACCTTGTATT<br>GTAGCGAGATGTAGCTGTGATACAGTCTGGAAAGCACGTGTCATATAGACAGGCAGCTAATGAATCTCACACTCTGGAACTGGACACCTTTGGTTCCAATTCTGGCCCTACCACTCTCGAGCTGTGGAAGAACTGC<br>ACCTCTGTTTTCTCCTCTGTAAAATGGGTATAATAATGGCACCAACCTCAGAGGGCTGCTGTGAGGGTGAAATAAGGGGATAATGGTATGATCTAGTTCACGGGTTTGTCGTAATGAGCTATTCTATGTGAAGTGCAG<br>GAAAACGTGCCTGGCATAGAGAATGTGAGCTGTCATGTGGGTGGCCCAGGTGGAGAGACTTGTCCCAAGCCTGGTTGGTCTTTGAACGTGTAGCCTGGCCTGTGACTGCTTACCTAAAATCTCCCCGCTTTTCCC<br>ACCTTCTCCTCTCTGATATGCCTGTTTGACATAGGTGAGATTGGATAGATCAAGGTGTTCAGGGCCTCTAGGCAGGGATGGGACTGTGGGCATGAGGACCAGGACCCAACCCCTCAGCCCCACTCTCCACCCCAG<br>GCTTTCTGAGCCATCCATCCTTCCCAAGAAACTGACAGAGCCACCCTGCCACTGGTACCAGCATTGACACCCATCTAGAGGTCCGAGGGGCAGCCCCAGGGCAGAGGAGATTTTGAGAGCCCACACCCCTGAC<br>CTGAGTGGGGAGGGGTTGAGCCTCTGGGCCACAGACCGCAGGTGCTGACTCATGGCCTCTGCTGGGCGATATAAAGGAACCAGCCCAGGGGCTTCCTACAGCCAGAGGGACGAGCTAGCCCGACG
2	**GC238-*PADI4*-Mut1**	CAACAAAAACAAACAAACAAAAA…….AAA**TACATATGAA**TAT……GACGAGCTAGCCCGACG
3	**GV238-*PADI4*-Mut2**	CAACAAAAACAAACAAACAAAAA……GGA**TACATATGAA**GGC……GACGAGCTAGCCCGACG
