# Supplementary material for: Helicobacter pylori upregulates PAD4 expression via stabilising HIF-1α to exacerbate rheumatoid arthritis
Source: Ann Rheum Dis. 2024 Aug 6;83(12):e225306. doi: 10.1136/ard-2023-225306 (PMC11671999; doi:10.1136/ard-2023-225306)
Supplement: online supplemental file 13 [file ard-83-12-s013.pdf]

Supplementary Table 5 HIF-1α shRNA sequences

	Gene	Target sequence
**1**	shRNA Control	TTCTCCGAACGTGTCACGT
**2**	sh-HIF-1α #1	GTGATGAAAGAATTACCGAAT
**3**	sh-HIF-1α #2	CAGCTGACCAGTTATGATTGT
**4**	sh-HIF-1α #3	AATGTGAGTTCGCATCTTGAT
