# Supplementary material for: Helicobacter pylori upregulates PAD4 expression via stabilising HIF-1α to exacerbate rheumatoid arthritis
Source: Ann Rheum Dis. 2024 Aug 6;83(12):e225306. doi: 10.1136/ard-2023-225306 (PMC11671999; doi:10.1136/ard-2023-225306)
Supplement: online supplemental file 14 [file ard-83-12-s014.pdf]

Supplementary Table 6 The primer sequences used for ChIP-qPCR assays

***PADI4* gene site**	**Primer**	**Sequence (5’-3’)**
Site 1	Forward	CCTTGTATTGTAGCGAGATGTAGC
Site 1	Reverse	GTTCTTCCACAGCTCGAGAG
