# Supplementary material for: Helicobacter pylori upregulates PAD4 expression via stabilising HIF-1α to exacerbate rheumatoid arthritis
Source: Ann Rheum Dis. 2024 Aug 6;83(12):e225306. doi: 10.1136/ard-2023-225306 (PMC11671999; doi:10.1136/ard-2023-225306)
Supplement: online supplemental file 15 [file ard-83-12-s015.pdf]

Supplementary Table 7 Top ten Gene Ontology terms of upregulated genes in *H. pylori*-infected GES-1 cells

	ID	GO-Term	Gene Ratio	P value	Adjusted P value	gene ID
**1**	GO:0006979	response to oxidative stress	22/216	6.08E-09	8.94E-06	SOD2/PYROXD1/ZNF622/KDM6B/MCL1/TXNIP/SIRT1/BTG1/AREG/SESN2/EGFR/BNIP3/ERN1/ETV5/EPAS1/SLC7A11/IL1A/JUN/MCTP1/FOSL1/STC2/TNFAIP3
**2**	GO:0009991	response to extracellular stimulus	23/216	7.82E-09	8.94E-06	SOD2/KLF10/PRKAB2/TRIM25/MAP1LC3B2/SIRT1/MAP1LC3B/RRAGC/PMAIP1/FNIP1/SESN2/BNIP3/IFI16/ASNS/STC1/IL1A/ADM/DDIT3/FOSL1/SPINK1/GDF15/UPP1/STC2
**3**	GO:0031667	response to nutrient levels	22/216	1.04E-08	8.94E-06	SOD2/KLF10/PRKAB2/TRIM25/MAP1LC3B2/SIRT1/MAP1LC3B/RRAGC/PMAIP1/FNIP1/SESN2/BNIP3/IFI16/ASNS/STC1/IL1A/ADM/DDIT3/SPINK1/GDF15/UPP1/STC2
**4**	GO:0062197	cellular response to chemical stress	19/216	1.04E-08	8.94E-06	SOD2/PYROXD1/ZNF622/KDM6B/MYC/MCL1/SIRT1/SESN2/EGFR/BNIP3/ZFP36L1/ERN1/ETV5/EPAS1/SLC7A11/JUN/DDIT3/ERRFI1/TNFAIP3
**5**	GO:0042060	wound healing	21/216	2.61E-08	1.79E-05	EREG/ELK3/JMJD1C/ANO6/VEGFA/DGKE/IL6ST/SERPINE1/ITGA5/FGF2/ARL8B/TNFRSF12A/SDC4/DCBLD2/CLDN1/SLC7A11/IL24/SERPINE2/F3/IL1A/TNFAIP3
**6**	GO:0071496	cellular response to external stimulus	17/216	1.13E-07	6.46E-05	KLF10/PRKAB2/MAP1LC3B2/SIRT1/MAP1LC3B/MAP3K2/ANKRD1/RRAGC/PMAIP1/FNIP1/SESN2/BNIP3/IFI16/ASNS/GADD45A/FOSL1/UPP1
**7**	GO:0045766	positive regulation of angiogenesis	13/216	2.04E-07	8.75E-05	VEGFA/SERPINE1/SIRT1/BTG1/ITGA5/FGF2/GATA6/NRP1/ETS1/F3/IL1A/ADM/HK2
**8**	GO:1904018	positive regulation of vasculature development	13/216	2.04E-07	8.75E-05	VEGFA/SERPINE1/SIRT1/BTG1/ITGA5/FGF2/GATA6/NRP1/ETS1/F3/IL1A/ADM/HK2
**9**	GO:0042594	response to starvation	13/216	4.50E-07	0.000172	KLF10/MAP1LC3B2/SIRT1/MAP1LC3B/RRAGC/PMAIP1/FNIP1/SESN2/IFI16/ASNS/ADM/DDIT3/UPP1
**10**	GO:0045765	regulation of angiogenesis	17/216	5.36E-07	0.000184	VEGFA/EPHA2/SERPINE1/SIRT1/BTG1/SPRED1/ITGA5/FGF2/GATA6/NRP1/ETS1/F3/IL1A/GADD45A/ADM/HK2/TNFAIP3
